# Experimental investigation on a thermochemical seasonal sorption energy storage battery utilizing MgSO_4_-H_2_O

**DOI:** 10.1007/s11356-023-28875-1

**Published:** 2023-08-23

**Authors:** Mostafa M. Salama, Sherif A. Mohamed, Mohamed Attalla, Ahmed N. Shmroukh

**Affiliations:** 1 Mechanical Engineering Department, Sohag University, Sohag, Egypt; 2 Department of Mechanical Engineering, South Valley University, Qena, 83521 Egypt

**Keywords:** Energy storage density, Water vapor sorption, Solid–gas reaction, Composite materials, Expanded natural graphite

## Abstract

Thermochemical sorption energy storage (TSES) is the most recent thermal energy storage technology and has been proposed as a promising solution to reduce the mismatch between the energy supply and demand by storing energy for months in form of chemical bonds and restore it in form of synthesis chemical reaction. Compared with sensible/latent thermal energy processes, TSES system has major advantages, including a high energy storage capacity/density and the possibility of long-term energy retention with negligible heat loss. Therefore, a solid–gas thermochemical sorption battery is established and investigated utilizing a composite working pair of MgSO_4_–H_2_O based on room temperature expanded graphite (RTEG), treated with sulfuric acid (H_2_SO_4_) and ammonium persulfate ((NH_4_)_2_S_2_O_8_) as a porous additive. The experimental results showed that energy storage density and sorption efficiency increase with the increment of charging temperature or decreasing of discharging temperature at a certain ambient temperature. Under experimental conditions, energy density ranged from 31.7 to 908.8 kJ/kg (corresponding to volume energy density from 11.7 to 335.8 MJ/m^3^), while sorption energy efficiency ranged from 28.3 to 79.1%. The highest values were obtained when charging, condensation, and discharging temperatures were 95, 20, and 15 °C, respectively. The maximum thermal efficiency was 21.1% at charging/discharging temperature of 95/15 °C with sensible to sorption heat ratio of 3:1.

**Graphical Abstract:**

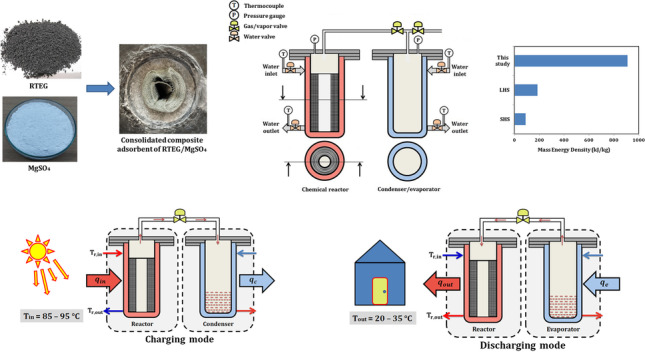

## Introduction

Energy is essential for human life development. However, the conventional energy resources, which are estimated to run out in several decades, are facing shortages, low efficiency, serious waste, and harmful emissions to the environment. Therefore, it is an inevitable trend to use renewable energy sources due to their large energy amounts and pollution-free. Solar energy is one of the promising and rapidly developing renewable energy technologies (Ye et al. 2022), as it has been widely researched and publicly accepted (Lin et al. 2021). Solar energy applications can be found in several aspects of our daily life, such as hot water supply, space heating, and cooking (Alva et al. 2017). The main disadvantage of solar energy is that it is an ambient source of energy. Solar irradiance varies with meteorological condition, location dependence, time, and season of a year. Thermal energy storage technology has been proposed as a promising solution to reduce the mismatch between the energy supply and demand by storing the energy from periods of high availability to periods of high demand or whenever needed (Zhang and Wang 2020).

Thermal energy storage can be divided into two main categories: long-term/seasonal storage and short-term storage, depending on the storage period. Moreover, it can be classified into thermochemical energy storage (TES), latent heat storage (LHS) utilizing phase change materials (PCMs), and sensible heat storage (SHS). However, thermochemical energy storage can be divided into two categories: thermochemical reaction heat storage (without sorption) and thermochemical sorption heat storage (ElBahloul et al. 2022).

**Fig. 1:**
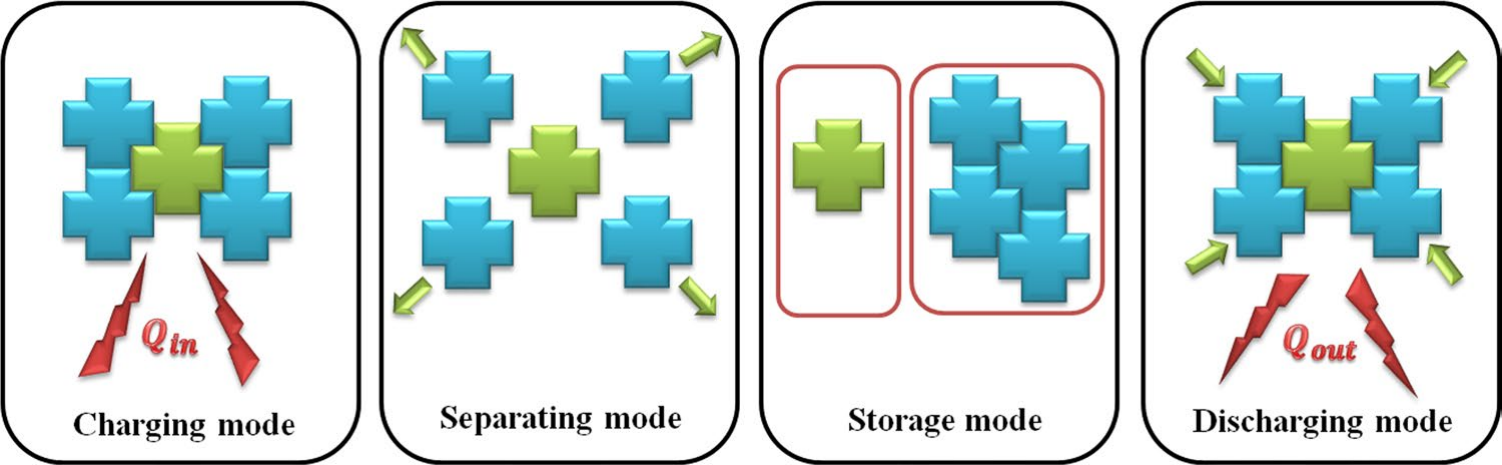
Solid–gas thermochemical sorption energy storage process

Compared with SHS and LHS processes, the thermochemical sorption energy storage (TSES) system has major advantages, including a high energy storage capacity, high storage density arising from the strong bonding force between sorption pairs, and the possibility of long-term energy retention with negligible heat loss once the sorbent and sorbate are separated (Zhang and Wang 2020), as well as the flexibility of stored temperature. Thermal energy storage has been emphasized as a critical technique for temporal and geographic decoupling of heat supply and demand, resembling a thermal battery in which heat is stored when it is not needed and is permitted to be transferred and released on demand by consumers. A water-based adsorption thermal battery (ATB) has piqued the interest of scientists as an appropriate solution to heat reallocation (Zeng et al. 2022). Solid–gas thermochemical sorption energy storage process is shown in Fig. 1. It mainly includes charging, separating, storage, and discharging modes.

In recent years, solid–gas thermochemical sorption energy storage has received great attention. Research on thermochemical materials has demonstrated considerable interest in thermochemical energy storage and heat transforming processes, which used in applications such as space heating, industrial heat recovery, and/or heat upgrade during the past 20 years. In previous years, reviews on salt hydrate reactions were published, such as Clark et al. (2020), Donkers et al. (2017), and Yan et al. (2015). Adsorption thermal battery (ATB) technology is now being commercialized and is being used in distributed space heating, industrial waste heat recovery, and smart phone thermal management. This technology will strive to broaden its use in domains connected to heat reallocation, energy saving, and emission reduction, such as greenhouse thermal management and ATB-based passive solar structures, in the future (Zeng et al. 2022). ATB is a new but underutilized technology that allows for new applications of renewable and waste heat (Strelova et al. 2023). MgSO_4_∙7H_2_O, SrBr_2_∙6H_2_O, Na_2_S∙9H_2_O, and MgCl_2_∙6H_2_O are the most investigated options. SrBr_2_∙6H_2_O is stable with appropriate energy density, but it is also the most costly one, while Na_2_S∙9H_2_O has the highest energy density but it is very corrosive. Other materials have lately been identified as extremely potential thermochemical media for construction applications; however, understanding these materials will require substantial more research (Palacios et al. 2020). Typically, the results of the hydration processes of these salts are thought to be higher hydrates with more crystal water molecules. In other circumstances, however, the RH pressure is so high that the hydration produces a saturated salt solution rather than a salt hydrate. Furthermore, MgSO_4_ has a high deliquescence relative humidity (DRH) of 90% at 30 °C, and it is a hydrothermally stable salt. Composite adsorption working pairs combine the benefits of chemical adsorbents and porous matrixes, to increase heat and mass transfer efficacy, while also enhancing the chemical adsorption stability (Wang et al. 2014). While consolidated composite sorbents based on EG are reported to have high thermal conductivity and permeability (Miao et al. 2021).

Li et al. (2016) suggested a solid–gas thermochemical sorption thermal battery and proved that it is a successful method for integrating short-term energy storage, long-term energy storage, and energy upgrade of solar thermal energy. They investigated that it has distinct advantages over conventional energy storage methods, including combined cold and heat storage, high energy density, and heat transformer, and can therefore help to promote solar space cooling and heating technologies. Li et al. (2017) developed a dual-mode thermochemical sorption energy storage system employing EG/SrCl_2_–NH_3_ for seasonal solar thermal energy storage. They demonstrated that when the heat output temperature is 35 °C and ambient temperature is 15 °C, the effective energy storage density is more than 700 kJ/kg, and the corresponding system efficiency reached about 41%. Xu et al. (2018a) presented a sorption thermal energy storage device for residential heating, consisted of newly designed zeolite 13X/MgSO_4_/ENG–TSA composite sorbent, with a salt mass fraction of 15%. It is tested and employed in the device. According to their experimental data, when charging and discharging temperatures were 250 °C and 25–90 °C, respectively. Their proposed device had an energy storage density of 120.3 kWh/m^3^. Moreover, Nguyen et al. (2022) developed a new two-component (composite) MgSO_4_/Hydroxyapatite (HAP) water sorbent, for sorption-based solar heat storage. HAP has a maximum water sorption capacity of 0.039 g/g. With varying MgSO_4_ concentrations, the composite of (20–MgSO_4_/HAP) has 3.7 times higher water sorption capacity. Their results demonstrated that the heat of hydration was 464 J/g for the HAP composite containing 20% MgSO_4_. Long-term cycling of dehydration at 150 °C and hydration at 30 °C with a relative humidity of 60% demonstrated the composite’s comparatively strong stability. The repeated stability of MgSO_4_–HAP is investigated, and it is discovered that this two-component sorbent is relatively stable after 20 dehydration/hydration cycles. Furthermore, Liu et al. (2022) prepared a novel salt composite, by combining various mesoporous silica with high pore size and volume with MgSO_4_. Salt composites produced at 30 °C, and a vapor pressure of 25 mbar demonstrated outstanding water adsorption capability of 50 wt% at a MgSO_4_ loading level of 50 wt%. According to their findings, the new composites were good choice for low-temperature energy storage. On the other hand, Bennici et al. (2022) developed a composite material of magnesium sulfate hydrates and activated carbon (MgSO_4_/AC) to increase the energy density. The hydration enthalpy increased with increasing MgSO_4_ concentration on activated carbon (AC), reaching a plateau for MgSO_4_ level more than 30 wt% (30–MgSO_4_/AC and 40–MgSO_4_/AC samples). The 30–MgSO_4_/AC hydration enthalpy increased from 859 J/g dry material at RH = 30% to 1324 J/g dry material at RH = 60%. The 30–MgSO_4_/AC sample was nearly steady after 8 cycles of hydration/dehydration. In addition, Zhao et al. (2022) created a K_2_CO_3_-based composite with about 85.7% salt concentration. The sorption properties were studied at various thicknesses, morphologies, and operating circumstances. The sorption rate of the K_2_CO_3_-based composite was found to be more than four times that of pure K_2_CO_3_, while both the mass and volume heat storage densities were found to be roughly 608.5 kJ/kg and 214.7 kWh/m^3^, respectively, indicating that this composite may be competitive in TES applications. Moreover, Zhao et al. (2016) combined SrBr_2_ and ENG–TSA, using different mass ratios, and the composite of 743 kg/m^3^ density containing 10% ENG–TSA performed well, with a high thermal conductivity of about 7.97 W/m∙K. Xu et al. (2018b) established a composite of MgSO_4_ impregnated zeolite 13X and activated alumina, to span both mid and low temperature ranges. The experimental energy storage densities were 123.4 kWh/m^3^ and 82.6 kWh/m^3^ for the zeolite 13X and the activated alumina composites, respectively. While the cyclability of sensible and latent heat storage systems is widely established, there is little research on the long-term stability of salt hydrates. The cyclability of a salt is determined by both the type of salt and how it is utilized. As a result, more study into cyclability into a variety of salts is required.

The overpopulation, intensive urbanization, excessive resource usage, and social inequality are the primary causes of the severe economic, environmental, technical, and social issues facing the building sector. In 2018, the building and construction sector was responsible for around 39% of the process-related carbon dioxide emissions. This is significant when considering greenhouse gas emissions and global climate change. The building sector may achieve carbon neutrality by 2050 or later or may continue to be carbon intensive, depending on the degree of current and future decarbonization of the building energy needs and of the power production system capacity dedicated to buildings which is approximately 50% now (Santamouris and Vasilakopoulou 2021). Building thermal energy storage capabilities and power-to-heat conversion technologies like heat pumps severely limit the degree of flexibility offered by building energy systems. It is crucial to record the dynamic response of the building energy system with thermal energy storage in order to quantify the flexibility of the building demand. In each circumstance of charging, discharging, or idle state, the instantaneous power flexibility demonstrates the potential power flexibility of TES and power-to-heat (Finck et al. 2018). As a result, a small size of TSES systems using adsorbent can provide about significant flexibility through the realization of thermal energy storage without heat losses. Therefore, TSES is a promising solution for building applications.

Based on previous literature, the studies using composite materials with a salt hydrate impregnated porous matrix mostly concentrated on how small samples hydrated utilizing a micro scale reactor. There is still much to learn in terms of understanding the mechanism and creating the sorption energy storage technology based on consolidated composite adsorbents. Further research is required, particularly on the impact of dehydration/hydration temperatures on the composite adsorbent hydration behavior and the hydration performance of a macro scale reactor using ENG composites. There is no research has been done on using magnesium sulfate (MgSO_4_) as adsorbent, room temperature expanded graphite (RTEG) as porous matrix, and water vapor (H_2_O) as adsorbate/refrigerant for energy storage applications. This study is aimed to develop an appropriate consolidated composite adsorbent based on RTEG for thermochemical sorption system and to look into its thermal and sorption properties. We need to understand the thermal effect of composite sorbent to know the time required for thermal equilibrium. Therefore, this proposed composite was selected for the proposed seasonal energy storage battery. One of the most often utilized hydrates is MgSO_4_, which has high adsorption capacity, due to the lowest molecular mass and the ability of 1 mol MgSO_4_ to complex 7 mol of H_2_O. MgSO_4_ has advantages of low price, availability, moderate equilibrium temperature, and high energy density. Moreover, water is one of the most prevalent refrigerants for adsorption systems. Battery utilizing hydrate offers benefits over the TSES battery based on ammoniates in terms of safety, affordability, and accessibility. Therefore, TSES utilizing working pair of RTEG/MgSO_4_–H_2_O is a promising and appropriate for driving batteries using low-grade energy sources, such as solar collector. The objectives of the proposed research are studying the thermal response of the new consolidated composite adsorbent based on RTEG, studying the thermal effects of reversible reactions between magnesium sulfate and water vapor, investigating the solid–gas thermochemical seasonal sorption energy storage battery utilizing composite working pair of RTEG/ MgSO_4_–H_2_O, since they are the most promising option for long-term energy storage in a low-temperature application, and studying the performance of the proposed TSES battery under various conditions including different charging and discharging temperatures. The results of this study prove the importance of TSES batteries in their use as a feasible application for building heat consumption.

## Working principle of thermochemical sorption energy storage battery

Chemical adsorption is a chemical reaction happens between adsorbent and adsorbate, and new types of molecules will be formed in the adsorption process. Commonly, the monolayer of the adsorbate will react with the chemical adsorbent, and after this reaction, the chemical adsorbent cannot adsorb more layers of molecules. The newly formed molecules will be decomposed in the desorption process (Wang et al. 2014).

The charging/discharging of magnesium sulfate occurs at temperatures ranging from 25 to 275 °C. Under varying temperatures, three phases of dehydration occurred. As illustrated in Eq. (1), the first step occurred at 25–55 °C and involved a 1-mol water loss. While regarding Eq. (2), the second stage occurred between 60 and 265 °C, resulting in a loss of around 5.9 mol of water. The last step occurred at 275 °C, resulting in a mass loss of 0.1 mol (the remaining water) as illustrated in Eq. (3). In the second and third phases of dehydration, little energy can be stored between 150 and 275 °C. As a result of heating to 150 °C, roughly 2.2 GJ/m^3^ is stored, making it a particularly promising material for compact seasonal storage. The first step hydration (∼ 25–30 °C) and initial dehydration stages overlap, and the conversion from MgSO_4_∙7H_2_O to MgSO_4_∙6H_2_O happens gradually when MgSO_4_∙7H_2_O is generated. The hydration/dehydration process of MgSO_4_/H_2_O working pairs can be represented by (van Essen et al. 2009):

1 $${\textrm{MgSO}}_4\bullet 7{\textrm{H}}_2{\textrm{O}}_{\left(\textrm{s}\right)}\leftrightarrow {\textrm{MgSO}}_4\bullet 6{\textrm{H}}_2{\textrm{O}}_{\left(\textrm{s}\right)}+{\textrm{H}}_2{\textrm{O}}_{\left(\textrm{g}\right)}$$

2 $${\textrm{MgSO}}_4\bullet 6{\textrm{H}}_2{\textrm{O}}_{\left(\textrm{s}\right)}\leftrightarrow {\textrm{MgSO}}_4\bullet 0.1{\textrm{H}}_2{\textrm{O}}_{\left(\textrm{s}\right)}+5.9{\textrm{H}}_2{\textrm{O}}_{\left(\textrm{g}\right)}$$

3 $${\textrm{MgSO}}_4\bullet 0.1{\textrm{H}}_2{\textrm{O}}_{\left(\textrm{s}\right)}\leftrightarrow {\textrm{MgSO}}_{4\left(\textrm{s}\right)}+0.1{\textrm{H}}_2{\textrm{O}}_{\left(\textrm{g}\right)}$$

According to the previous dehydration temperature required for the chain reaction, this research work studied the performance of MgSO_4_/H_2_O solid–gas thermochemical reaction for MgSO_4_(6/3), MgSO_4_(6/2.5), and MgSO_4_(6/2.2) which can be operated with solar energy at moderate temperatures of 60–85 °C as illustrated in Eq. (4), 60–90 °C as illustrated in Eq. (5), and 60–95 °C as illustrated in Eq. (6). The battery operates according to the following reversible reactions:

4 $${\textrm{MgSO}}_4\bullet 6{\textrm{H}}_2{\textrm{O}}_{\left(\textrm{s}\right)}+3\Delta {\textrm{H}}_{\textrm{r}}\leftrightarrow {\textrm{MgSO}}_4\bullet 3{\textrm{H}}_2{\textrm{O}}_{\left(\textrm{s}\right)}+3{\textrm{H}}_2{\textrm{O}}_{\left(\textrm{g}\right)}$$

5 $${\textrm{MgSO}}_4\bullet 6{\textrm{H}}_2{\textrm{O}}_{\left(\textrm{s}\right)}+3.5\Delta {\textrm{H}}_{\textrm{r}}\leftrightarrow {\textrm{MgSO}}_4\bullet 2.5{\textrm{H}}_2{\textrm{O}}_{\left(\textrm{s}\right)}+3.5{\textrm{H}}_2{\textrm{O}}_{\left(\textrm{g}\right)}$$

6 $${\textrm{MgSO}}_4\bullet 6{\textrm{H}}_2{\textrm{O}}_{\left(\textrm{s}\right)}+3.8\Delta {\textrm{H}}_{\textrm{r}}\leftrightarrow {\textrm{MgSO}}_4\bullet 2.2{\textrm{H}}_2{\textrm{O}}_{\left(\textrm{s}\right)}+3.8{\textrm{H}}_2{\textrm{O}}_{\left(\textrm{g}\right)}$$

where ∆H_r_ is the change in the standard enthalpy and theoretically equals 50.6 and 44.7 kJ/mol H_2_O for dehydration and hydration, respectively (van Essen et al. 2009).

**Fig. 2:**
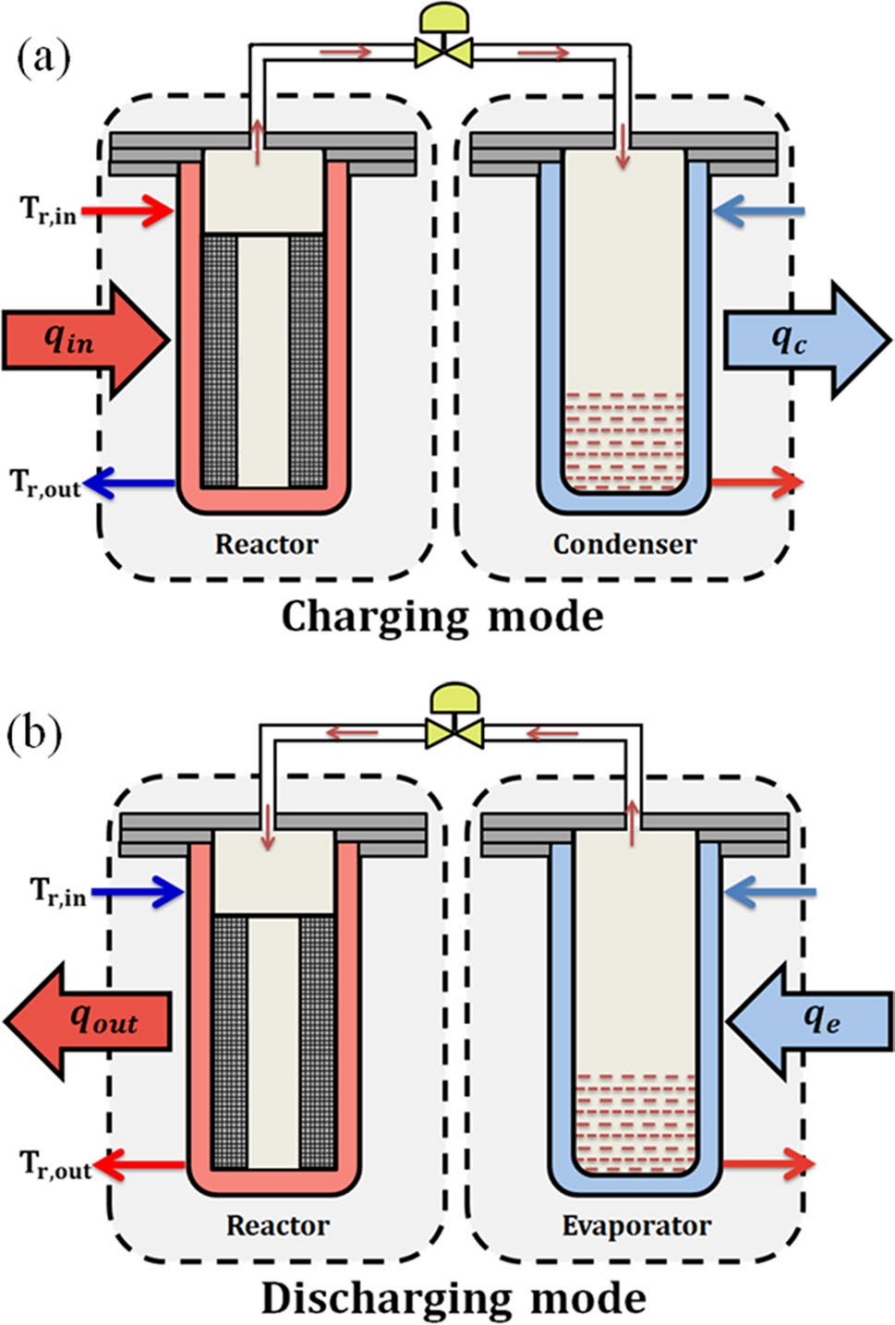
Schematic diagram of sorption storage battery. **a** Charging mode. **b** Discharging mode

Thermochemical sorption energy storage battery can be used for different applications: energy storage, energy upgrade, and combined heating and cooling. The current study focuses on energy storage which considered as the basis idea of thermochemical sorption process. Energy storage mainly has two processes: charging/dehydration process and discharging/hydration process. Figure 2 illustrates the schematic diagram of the thermochemical sorption processes. The battery mainly contains reactor, condenser/evaporator, and pipeline with a valve. Figures 2a and b presented the charging and discharging modes, respectively.

During charging mode, as shown in Fig. 2a, the chemical reactor is heated by heat input (q_in_) with moderate temperature of 75–95 °C, which can be obtained from solar energy or waste heat source. Some of heat input used as sensible heat for the reactor and composite adsorbent, and then the heat breaks the bonds between salt and water. Now, water vapor can escape and flow through a connecting pipe between reactor and condenser, then it condensates to liquid water rejecting its condensation heat (q_c_). When the reactor inlet and outlet temperatures reach the same value or with little difference, this means that there is no more water will escape. Now the valve can be closed, and the heat is stored in the form of bonding energy with no heat losses.

During the discharging mode, as shown in Fig. 2b which works in the other direction, when the heat stored is needed even after months, the valve is opened again. The condenser now acts as an evaporator. Liquid water takes its evaporation heat (q_e_) and evaporates to the reactor to be adsorbed by the reactive salt. The hydration reaction occurs, and the heat releases as a heat output (q_out_).

## Manufacturing process of the proposed new TSES material

Porous heat transfer matrixes of composite/consolidated chemical adsorbents have recently been proposed. Expanded natural graphite (ENG) and ENG–TSA are the most common matrixes which have been used widely, and it can achieve promising results. For ENG–TSA, thermal conductivity and permeability perpendicular to the compression direction, was 50 times and 200 times higher than that of parallel compression direction, respectively (Wang et al. 2011).

**Fig. 3:**
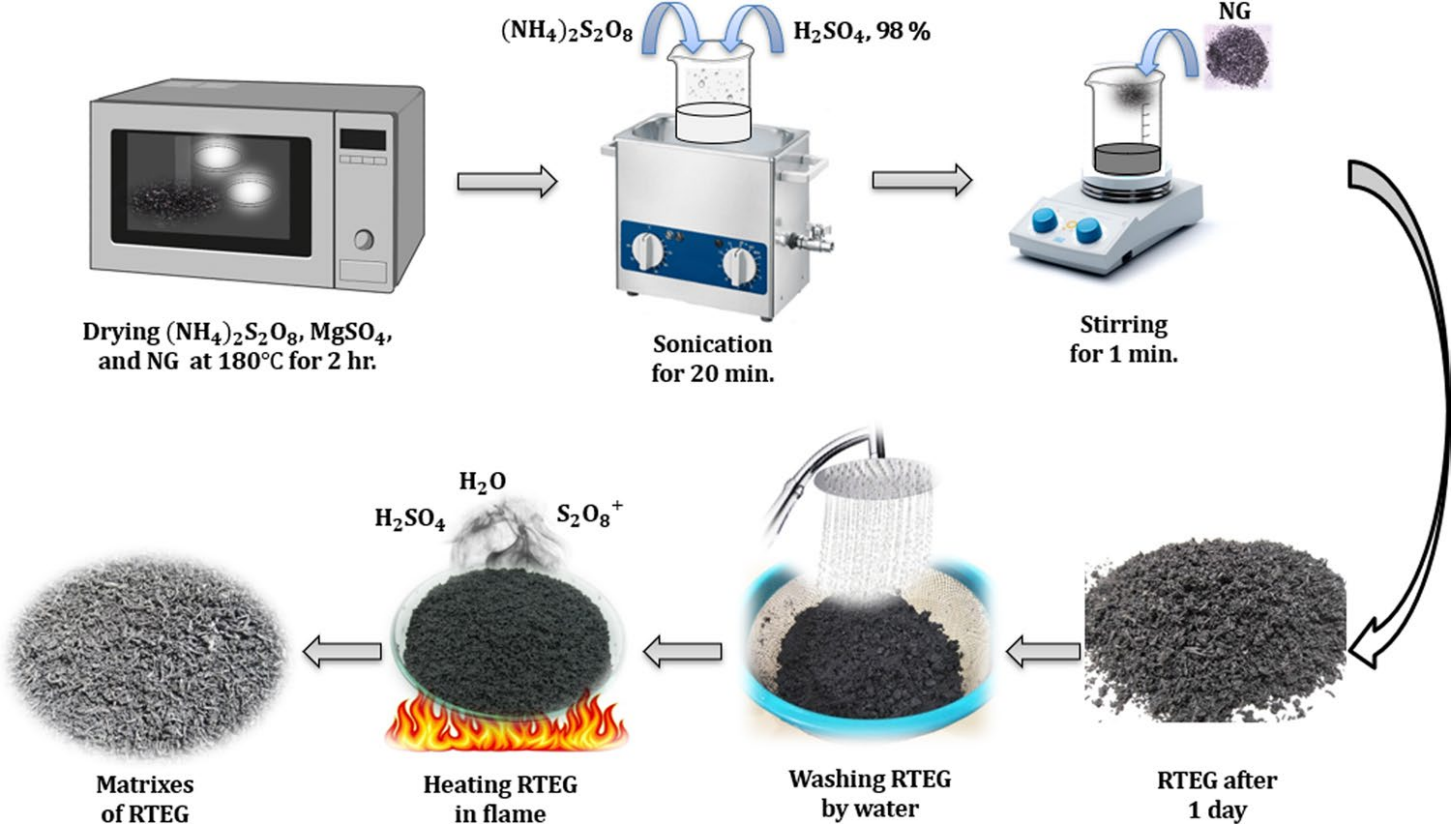
The procedures of expanding NG to RTEG

Here, the thermochemical sorption energy storage material is a solidified, consolidated composite sorbent based on room temperature expanded graphite (RTEG) impregnated with magnesium sulfate (MgSO_4_). Liu et al. (2017) proposed a simple, effective method, more energy-conserving, and pollutant-free approach to prepare expanded graphite, in which the intercalation and expansion of graphite are realized by only one step under ambient conditions. To prepare 1 g of expanded graphite, a combination of 5 g of (NH_4_)_2_S_2_O_8_ and 3 ml of concentrated H_2_SO_4_, 98% are sonicated for 5 min. Then, under ambient circumstances, 1 g of natural graphite (NG) was added to the resulting mixture and stirred for 30 s, yielding a slurry of (NH_4_)_2_S_2_O_8_, H_2_SO_4_, and NG. The slurry was changed into expanded graphite after just standing for a period of time at room temperature. The resulting RTEG is washed by water and then heated in a flame to remove H_2_SO_4_ or any other residue followed by a drying in an oven at 300 °C for 2 h. The final bulk density of RTEG can reach to about 10 kg/m^3^. Figure 3 shows the procedures of expanding 15 g of NG to RTEG.

**Fig. 4:**
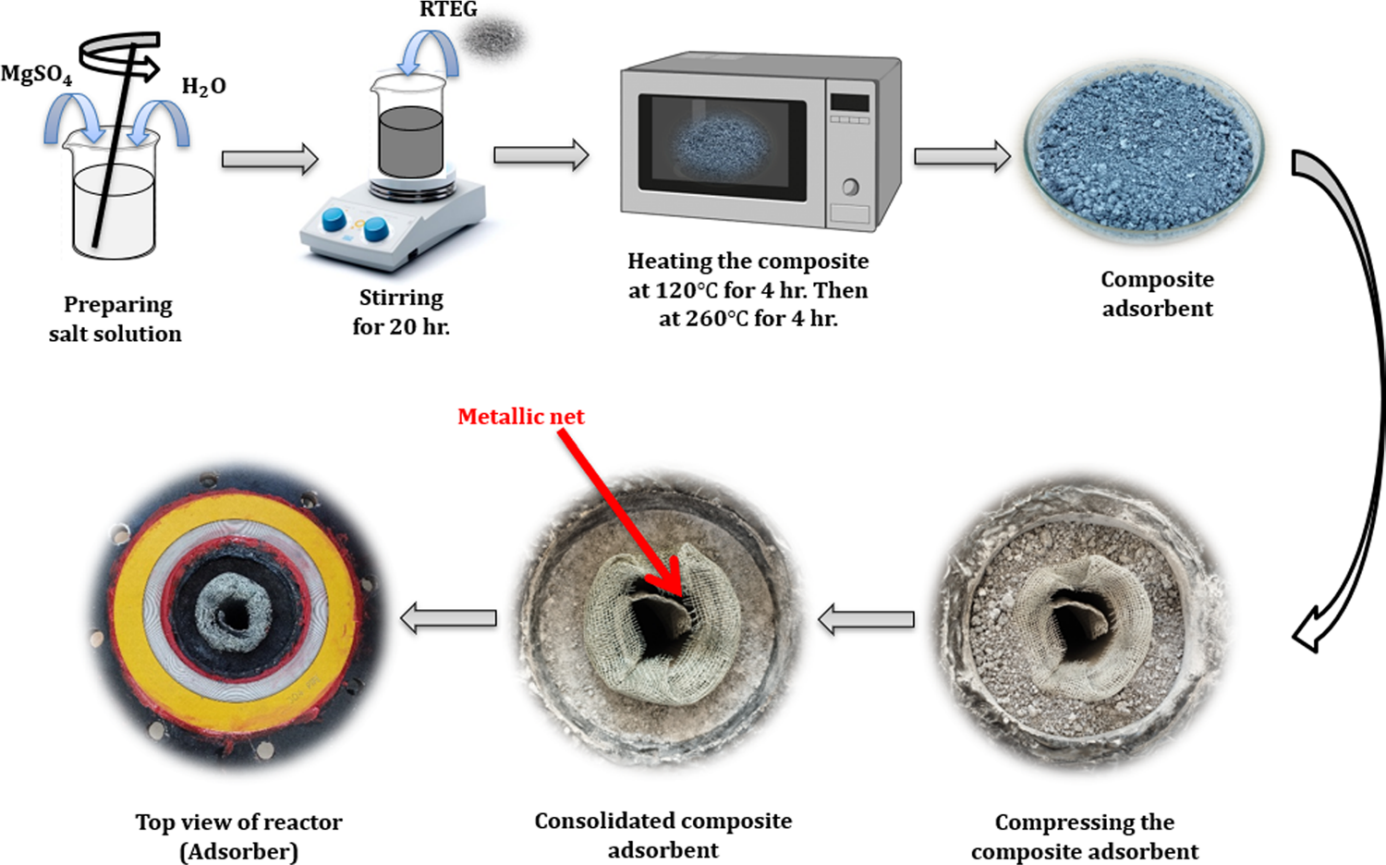
The main sequence of manufacturing process of composite sorbent of RTEG/MgSO_4_

Figure 4 shows the main sequence of manufacturing process of composite sorbent of RTEG/MgSO_4_. The pure salt is used in powder form. The procedures of impregnation are the following: firstly, MgSO_4_ is dried at a constant temperature of 180 °C in an electrical oven for 2 h. Secondly, the salt is dissolved in distilled water to make a salt solution of 16% concentration. Thirdly, a stirrer device is used to stir the mixture of salt solution and RTEG, and lastly, the composite is dried at 120 °C for 4 h to remove water followed by another 4 h at 260 °C to remove crystal water. The resulting composite density ranges of 75–100 kg/m^3^. The composite sorbent is compressed to form consolidated composite sorbent of 370 kg/m^3^ bulk density.

## Performance evaluation

During the charging/discharging phase, the instant input/output power (P) can be described as:

7 $$\textrm{P}=\dot{\textrm{m}}\bullet {\textrm{C}}_{\textrm{p}}\bullet \Delta \textrm{T}$$

where $\dot{\textrm{m}}$ (kg/s) and C_p_ (kJ/kg∙K) are the mass flow rate and the specific heat of heat transfer fluid, respectively. While, ∆T (°C) is the difference between inlet and outlet temperatures of the heat transfer fluid of the chemical reactor.

The specific heat capacity of reactor body (c_p_) can be determined from the heat absorbed as:

8 $$\textrm{Q}=\textrm{m}\bullet {\textrm{C}}_{\textrm{p}}\bullet \Delta \textrm{T}$$

where Q (kJ) is the total heat absorbed, m (kg) is the mass of the reactor body, and ∆T (°C) is the difference of reactor body temperatures.

During the energy storage stage (charging phase/dehydration), the total heat input of the thermochemical energy storage battery (Q_in_) can be calculated by:

9 $${\mathrm Q}_{\mathrm{in}}={\mathrm Q}_{\mathrm{in},\mathrm{sens}}+{\mathrm Q}_{\mathrm{in},\mathrm{sorp}}=\sum\nolimits_{\mathrm i=0}^{\mathrm n}\dot{\mathrm m}\cdot{\mathrm C}_{\mathrm p}\cdot{\left({\mathrm T}_{\mathrm r,\mathrm{in}}-{\mathrm T}_{\mathrm r,\mathrm{out}}\right)}_{\mathrm i}\Delta\mathrm t$$

During the energy release stage (discharging phase/hydration), the total heat output of the thermochemical energy storage battery (Q_out_) can be calculated by:

10 $${\mathrm Q}_\text{out}={\text{Q}}_{\text{out},\text{sens}}+{\text{Q}}_{\text{out},\text{sorp}}=\sum\nolimits_{\text{i}=0}^\text{n}\dot{\text{m}}\cdot{\text{C}}_\text{p}\cdot{\left({\text{T}}_{\text{r},\text{out}}-{\text{T}}_{\text{r},\text{in}}\right)}_\text{i}\Delta\text{t}$$

where Q_sens_ (kJ) is the sensible heat of metallic part and the composite sorbent of the chemical reactor. Q_sorp_ (kJ) is the reaction heat of chemical reactive salt (chemical dehydration/hydration heat). T_r,in_ and T_r,out_ (°C) are the inlet and outlet temperature of heat transfer fluid of the chemical reactor. ∆t (s) is the time interval for every two-scanning data. n is the scanning times.

The ratio of sensible and sorption heat can be calculated as:

11 $$\upvarepsilon =\frac{{\textrm{Q}}_{\textrm{sens}}}{{\textrm{Q}}_{\textrm{sorp}}}$$

The mass and volume energy storage densities of the consolidated composite material (ED_m_ and ED_V_) are defined as the ratio of total heat output to the mass and filling volume of composite sorbent, respectively, and can be calculated as:

12 $${\textrm{ED}}_{\textrm{m}}=\frac{{\textrm{Q}}_{\textrm{out}}}{{\textrm{m}}_{\textrm{cs}}}$$

13 $${\textrm{ED}}_{\textrm{V}}=\frac{{\textrm{Q}}_{\textrm{out}}}{{\textrm{V}}_{\textrm{cs}}}$$

where m_cs_ (kg) and V_cs_ (m^3^) are the mass and filling volume of composite sorbent inside the chemical reactor, respectively.

Sorption efficiency (ɳ_sorp_) is defined as the ratio of the effective heat output during the discharge phase to the sorption heat input during the charge phase of the chemical reactor and can be calculated as:

14 $${\upeta}_{\textrm{sorp}}=\frac{\textrm{Effective}\ \textrm{sorption}\ \textrm{heat}\ \textrm{output}}{\textrm{Total}\ \textrm{sorption}\ \textrm{heat}\ \textrm{input}}=\frac{{\textrm{Q}}_{\textrm{out},\textrm{eff}}}{{\textrm{Q}}_{\textrm{in},\textrm{sorp}}}$$

Thermal efficiency (ɳ_th_) is defined as the ratio of the effective heat output during the discharge phase to the total heat input during the charge phase of the chemical reactor and can be calculated as:

15 $${\upeta}_{\textrm{th}}=\frac{{\textrm{Q}}_{\textrm{out},\textrm{eff}}}{{\textrm{Q}}_{\textrm{in}}}$$

## Experimental procedure

**Fig. 5:**
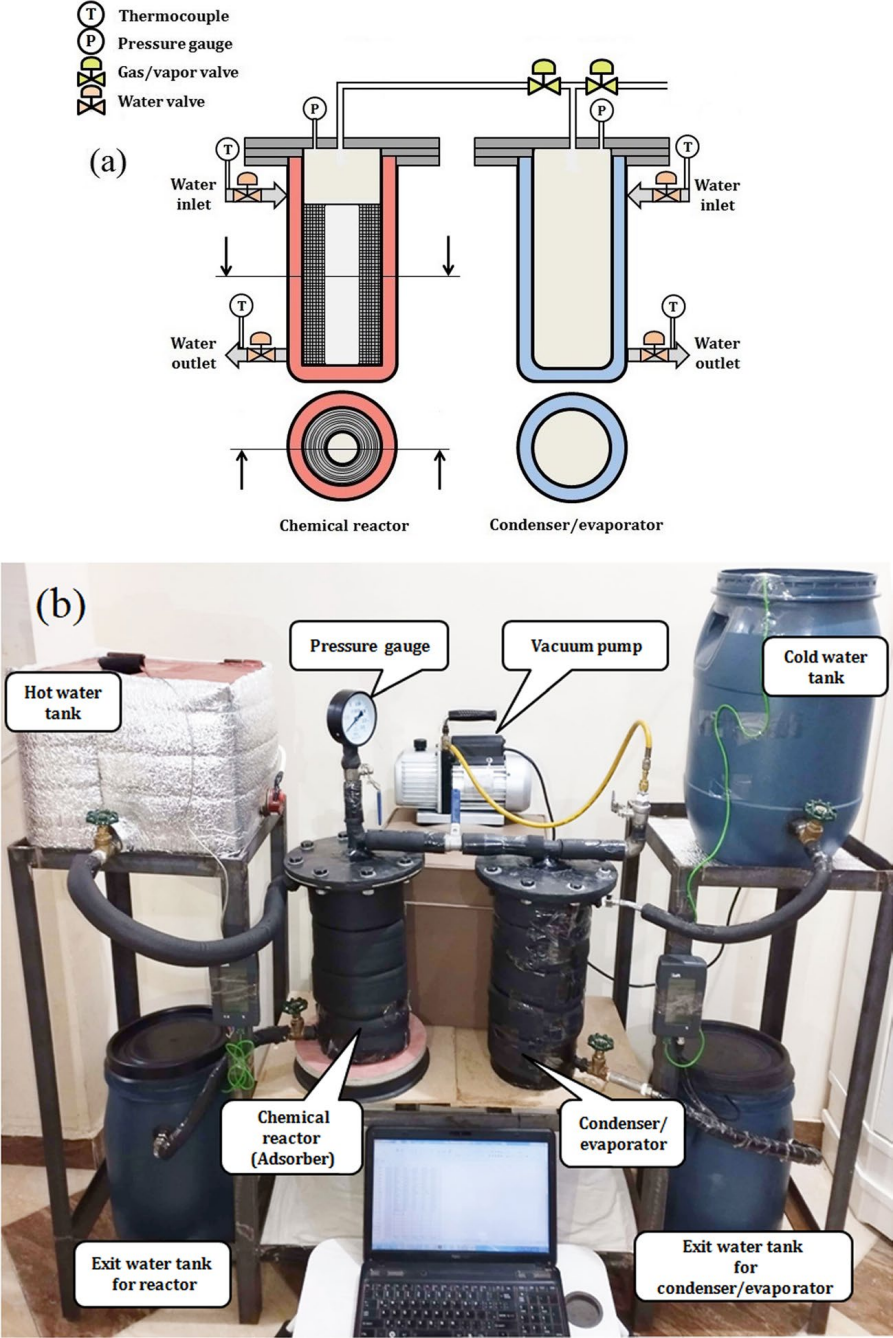
Thermochemical sorption battery system. **a** Schematic diagram. **b** Experiment layout

The layout of the sorption battery system is shown in Fig. 5. Experimental apparatus includes chemical reactor, condenser/evaporator, water tanks, vacuum pump, several thermocouples, pressure gauge, gas valves, water valves, flexible hose, and some other accessories. Jacket type heat exchanger is used as chemical reactor and condenser/evaporator (condenser in case of charging mode and evaporator in discharging mode). Water tanks are used to control the temperature of consolidated composite sorbent in the reactor. Heating and/or cooling water is circulating heat exchange fluid. Water is available and has high specific heat capacity of 4.18 kJ/kg∙K. The consolidated composite sorbent of magnesium sulfate MgSO_4_ impregnated into expanded graphite RTEG, with a mass of 460 g in 520 g of total composite material, and it is compressed to 370 kg/m^3^ in the chemical reactor. Distilled water of 650–700 ml is put inside the condenser/evaporator.

To get more accuracy, it is important to lower the time interval between every two-scanning data. So, the time interval for measuring the parameters is chosen to be every 2 min. Four thermocouples with K-type (range from −50 to 150 °C with tolerance of ± 0.1%) are used to measure required temperatures (inlet and outlet of heat exchange fluid). The values of measured temperatures are displayed on two data logger screens. Pressure gauge of Bourdon gauge type (full scale from 0 to 0.15 MPa abs with tolerance of ± 1%) is placed in the reactor to measure the battery pressure. During charging, the readings of pressure are recorded for the reactor when the intermediate valve is closed, while the pressure of condenser is maintained constant at the initial pressure. When the intermediate valve is opened, the pressure readings are for whole batter. During discharging, all readings of pressure are for both of reactor and condenser as the intermediate valve is always opened. Water flow rate is already controlled by valves before the starting and measured using a beaker and stop watch. The vacuum pump is used in times of need to maintain vacuum inside.

During dehydration/hydration, the input/output heat quantities are calculated according to inlet and outlet temperatures as well as the water mass flow rate and its specific heat as in Eqs. (9 and 10). The total heat is the sum of heat quantities calculated for every two-scanning data of 2 min.

For dehydration, the sensible input heat can be calculated according to Eq. (8) with the aim of knowing the start-up temperature, final temperature, the total mass, and the sensible heat capacity of the reactor body. Hence, the sorption input heat is the difference between the total input heat and sensible input heat. For hydration, the sensible recovery heat and sorption heat are separated. During long-term storage, only sorption heat recovery is considered.

Knowing the mass and filling volume of composite sorbent inside the chemical reactor, the energy storage densities can be calculated according to Eqs. (12 and 13). The mass and filling volume of sorbent is 520 g and 1407.3 cm^3^, respectively.

The performance of TSES battery is studied with different conditions including the response of the new consolidated adsorbent as well as short-term sensible and sorption energy storage and long-term/seasonal sorption energy storage. The following experiments were carried out to investigate and understand the performance of the proposed TSES battery.

### Thermal response of composite RTEG/MgSO_4_ during sensible charging

**Fig. 6:**
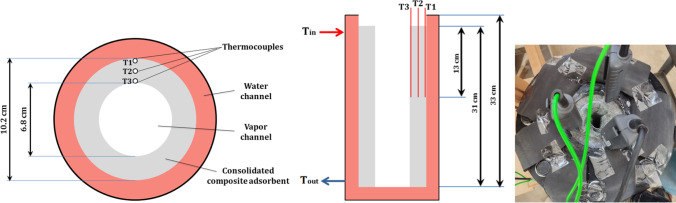
Places of thermocouples inside the consolidated composite adsorbent in reactor

The main goal of this experiment is to study the response of the new composite sorbent to the heat and analyze the temperature profile through its radial direction inside the reactor. Three thermocouples are placed inside the sorbent, which cover the whole thickness as shown in Fig. 6. One of them (denoted by T1) is placed where touch the first particles that touch the reactor wall from inside. The second (denoted by T2) is placed in between in the mid. While, the third one (denoted by T3) is placed where touch the final particles that touch the metal mesh in the bore. All were inserted inside the composite salt with depth of 13 cm from the top surface. Two thermocouples were used to measure the inlet and exit reactor water temperatures. Temperatures were plotted versus time. To understand better, the power absorbed by the reactor, including the composite material inside, is calculated according to the inlet and exit water temperatures and plotted versus time. During this sensible heat storage experiment, total heat absorbed and specific heat of reactor body were calculated. The results are important to determine the sorption heat as illustrated in discussion section.

### Thermal response of composite RTEG/ MgSO_4_ during sensible discharging

To study the response of the consolidated composite adsorbent during discharging, a reverse experiment is done where cold water at 35 °C flow inside the chemical reactor. Now, the sensible heat stored can be released. The positions of thermocouples were the same. The temperature profiles of the three different thermocouple positions as well as inlet and exit water temperatures were plotted with time. The output power was calculated according to the inlet and exit water temperatures and plotted versus time. During this sensible heat recovery experiment, total heat released was measured.

### Charging of TSES battery

To study the performance of the sorption battery, two different sorption experiments were done: a short-term storage experiment with dehydration temperature of 85 °C and a long-term/seasonal storage experiment with dehydration temperature of 90 °C. It is worth to be mentioned that sorption energy cannot separate from sensible heat during charging/dehydration, since the water vapor escaped from salt takes place gradually with temperature. The sorption heat can be determined from the difference between total input heat during charging and the calculated sensible heat.

For short-term storage, the reactor temperature was controlled by hot water, with a range of 83.8–86.1 °C, with average dehydration temperature of 85 °C. Valve between reactor and condenser was opened when the exit water temperature raised to 69 °C, as this is the minimum dehydration temperature according to van Essen et al. (2009). Water vapor escaped from sorbent (reactive salt) and condensed in the condenser, which controlled by ambient water temperature of 25–26 °C. Inlet and exit water temperatures plotted with time. Instant power was calculated and plotted with time including instant power per 1 kg of composite adsorbent. The total charging heat also was calculated.

For long-term/seasonal storage, the reactor temperature was controlled by hot water with average dehydration temperature of 90 °C. The valve, in this case, was opened when the exit water temperature raised to 81 °C when the reactor pressure reached 26 kPa. The condenser controlled by ambient water temperature of 21–22 °C. Inlet and exit water temperatures as well as pressure plotted with time. Instant power was calculated and plotted with time including instant power per 1 kg of composite adsorbent. The total charging heat also was calculated.

### Discharging of TSES battery

For short-term storage, discharging mode divided into sensible discharging and sorption discharging. During sensible discharging, the valve was closed, and the reactor supplied with discharging temperature of 27.4 °C. Inlet and exit water temperatures and instant output power were plotted versus time. The stored sensible heat was calculated. Hence, the thermal efficiency of sensible energy storage was calculated. During sorption discharging, the valve was opened to allow water vapor to react with salt in the reactor. The evaporator was controlled by the ambient or warm water according to the equilibrium pressure, and then H_2_O evaporated and took its way to the reactor to be adsorbed by the reactive salt (MgSO_4_∙3H_2_O). The hydration reaction occurred, and the heat is released, to be taken by discharging/ambient temperature. After that, the hydration reaction heat released was calculated.

For long-term/seasonal storage, sorption discharging mode only was considered. Inlet and exit water temperatures and pressures were plotted versus time. The evaporator was controlled by the ambient or warm water according to the equilibrium pressure, and then H_2_O adsorbed by the reactive salt (MgSO_4_∙2.5H_2_O).

### Performance of TSES battery at different charging and discharging temperatures

For different charging/dehydration and discharging/hydration temperatures, 9 groups of experiments were carried out to investigate the performance of TSES battery. During charging mode, the chemical reactor was controlled with hot water temperature ranged of 75–95 °C, i.e., 75, 80, 85, 90, and 95 °C. Condensation and discharging temperatures adjusted according to ambient conditions.

During discharging mode, the chemical reactor was controlled with cold water temperature ranged of 15–30 °C, i.e., 15, 20, 25, and 30 °C. Charging temperature was chosen at 95 °C, while the condensation process took placed at ambient temperature of 20 °C

In this section, only the sorption/hydration energy has been considered without considering the sensible heat recovery. As a result, long-term/seasonal energy storage performance of TSES battery has been discussed. For all experiments, mass and volume energy densities were calculated. Sorption and thermal efficiencies of effective heat released also were calculated and discussed.

### Error analysis

Errors for propagating uncertainties through calculations are determined by using the following general method (Shmroukh 2019; Mohammed et al. 2021, 2022):

16 $$\delta R=\sqrt{{\left[\frac{\partial R}{\partial {x}_1}\delta {x}_1\right]}^2+{\left[\frac{\partial R}{\partial {x}_2}\delta {x}_2\right]}^2+\dots +{\left[\frac{\partial R}{\partial {x}_N}\delta {x}_N\right]}^2}$$

Based on accuracies of measured parameters, as listed in Table 1, the whole uncertainty analysis is presented in Table 2.

**Table 1:** Error analysis of the measured parameters

Variable	Symbol	Error (%)
Temperature	T	± 0.1
Pressure	p	± 1
Water flow rate	ṁ	± 1.5

**Table 2:** Error analysis of the determined parameters

Parameter	Symbol	Error (%)
Power	P	± 1.4
Heat	Q	± 2.8
Energy density	ED	± 2.8
Sorption efficiency	η_sorp_	± 5.1
Thermal efficiency	η_th_	± 3.7

## Results and discussions

### Temperature profiles of composite RTEG/MgSO_4_ during sensible charging

**Fig. 7:**
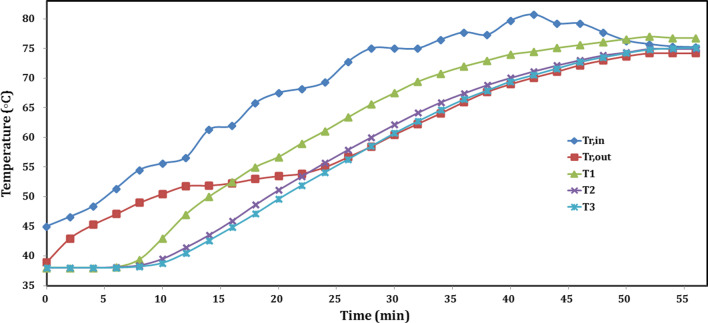
Temperature profiles of consolidated composite adsorbent during sensible charging

Figure 7 shows the temperature profiles of composite sorbent, as well as the inlet and outlet reactor temperatures during charging. Hot water temperature started at 45 °C, while the composite was at ambient temperature of 38 °C. Inlet water is allowed to rise as shown in Fig. 7 and reached 80.7 °C after 42 min. The exit temperature increased with time and started its stable increment after 22 min. The recorded composite salt temperature was larger than the exit water temperature; this has been interpreted as the heat stored in the composite salt, which required more time to transfer to the exit water. The temperature at position 3 (T3) was also larger, to ensure that the whole composite stored the heat. This will help us understanding more in the following sorption experiment where the exit water temperature will be always equal to or slightly less than the composite salt. At the beginning, the increment of composite temperature was very low, since a lot of heat added is absorbed by the metallic components of the reactor. After 6 min, the composite material began to response to heat, and the temperature increased with time. It was expected to see that T1 was the highest and T3 is the lowest one. The differences between the three temperatures were decreased with time, and the difference between T1 and T2 is more than that for T2 and T3. The temperatures of T1, T2, and T3 were 43, 39.5, and 38.8 °C, respectively, after 10 min. Then, after 44 min from the start, the three temperatures raised up to about 75.1, 72.1, and 71.6 °C, respectively. Moreover, the differences decreased more where T2 and T3 could be the same and T1 slightly larger. While after 52 min, it could be investigated that the whole material reaches the equilibrium with the reactor since the temperatures did not change and be constant at 77, 74.9, and 74.8 °C for T1, T2, and T3, respectively. The final mean temperature value for the material was about 75 °C, in case of heating most of time with mean temperature of 77 °C. It is outstanding for the consolidated composite sorbent to reach its equilibrium after only 52 min.

### Instant input power profile of sensible heat storage during sensible charging

**Fig. 8:**
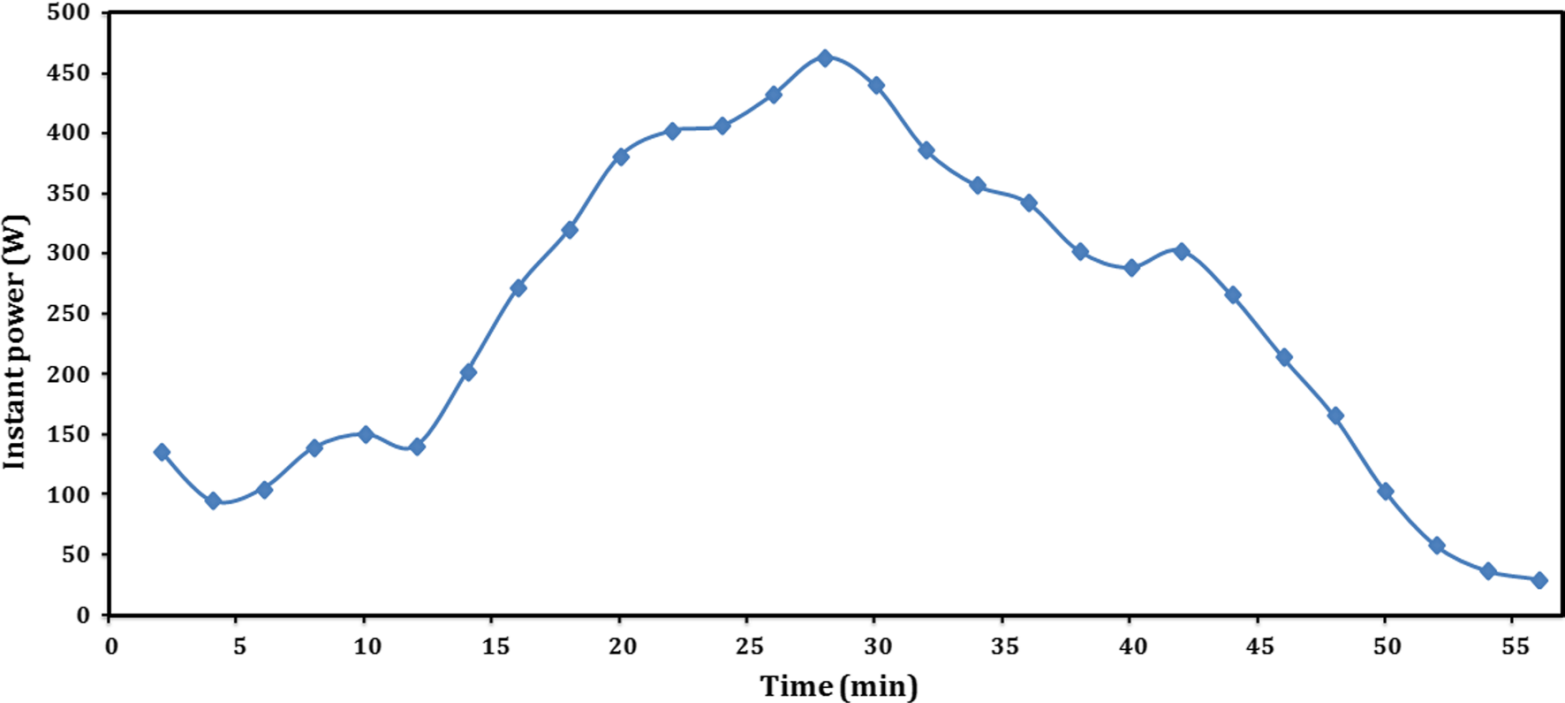
Instant sensible power absorbed by reactor body during sensible charging

The input power absorbed was plotted versus time as shown in Fig. 8. The power increases with the increase of temperature difference between inlet and exit temperatures of heating fluid. As illustrated previously, the temperature difference between inlet and exit temperatures can estimate the instant power absorbed and predict the trend of power curve. As this difference decreased largely through the first 4 min, the corresponding power decreased from 136.4 W after 2 min to 95.2 W after 4 min. After that, the power increased to 150.6 W after 10 min, followed by another slightly decreasing to 140.7 W after 12 min. From 12 to 28 min, the power increased gradually until reaching its highest value of 463.3 W. Although the inlet water temperature continued in increasing from 28 to 42 min, the exit water temperature also increased from 58.5 to 70.1 °C, as the reactor taking most of its heat. Therefore, the temperature difference is decreased, causing a decreasing in power from 463.3 to 302.7 W, followed by continuous decreasing up to its minimum value of 29.8 W at 56 min. Furthermore, after 56 min, the experiment of sensible heat storage could be finished with temperature increment from 38 to 75 °C with a difference of the reactor body temperatures of 37 °C, while the mean power value was 247.9 W, and the total input heat consumed during the sensible heat storage was 837.8 kJ. Knowing the total mass of the reactor body during sensible charging which was 14.5 kg, the sensible heat capacity of the reactor body could be determined using Eq. (8) and equals to 1.56 kJ/kg.

### Temperature profiles of composite RTEG/MgSO_4_ during sensible discharging

**Fig. 9:**
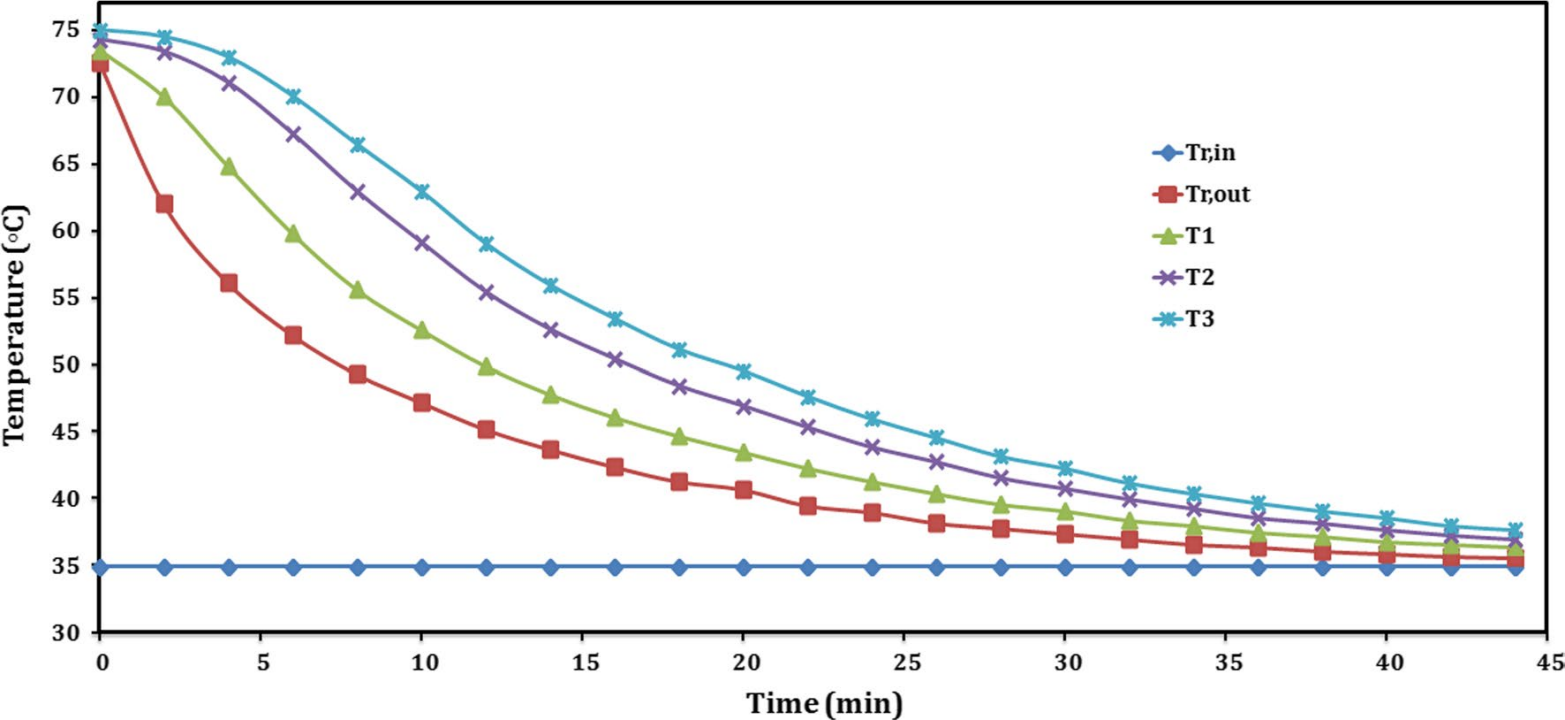
Temperature profiles of consolidated composite adsorbent during sensible discharging

During charging, the final reactor temperature was 75 °C. For discharging with water temperature of 35 °C as illustrated in Fig. 9, the exit water temperature decreased gradually with time, from 72.6 to 35.6 °C during 44 min. The decreasing was sharply at the first stage from 0 to 12 min, as high amount of the stored heat is released and followed by moderate decreasing, from 12 to 22 min as most of the stored heat is captured from cold water. After 22 min, the exit water temperature is partly decreased as shown in Fig. 9. While after 40 min, it started to be constant at 35.6 °C, with a temperature difference of about 0.6 °C only between inlet and exit temperatures, and the experiment was about to reach its end. According to that, the reactor needs extra 30 min to reach equilibrium, and this time was so large according to the actual discharging test of 44 min. During the extra 30 min, the released power was so small that it can be neglected compared with the total output power released. Partly similar trends resulted of T1, T2, and T3, according to the exit temperature trend where there were always a temperature differences between the three temperatures. The temperatures of T1, T2, and T3 at starting were 73.5, 74.3, and 75 °C, respectively, and decreased with time as the stored heat is released. While after 44 min, the temperatures of T1, T2, and T3 reached about 36.4, 37, and 37.7 °C. The final reactor temperature was about 36 °C. It is promising for the proposed new composite sorbent to release its stored energy during only 44 min.

### Instant output power profile of sensible heat recovery during sensible discharging

**Fig. 10:**
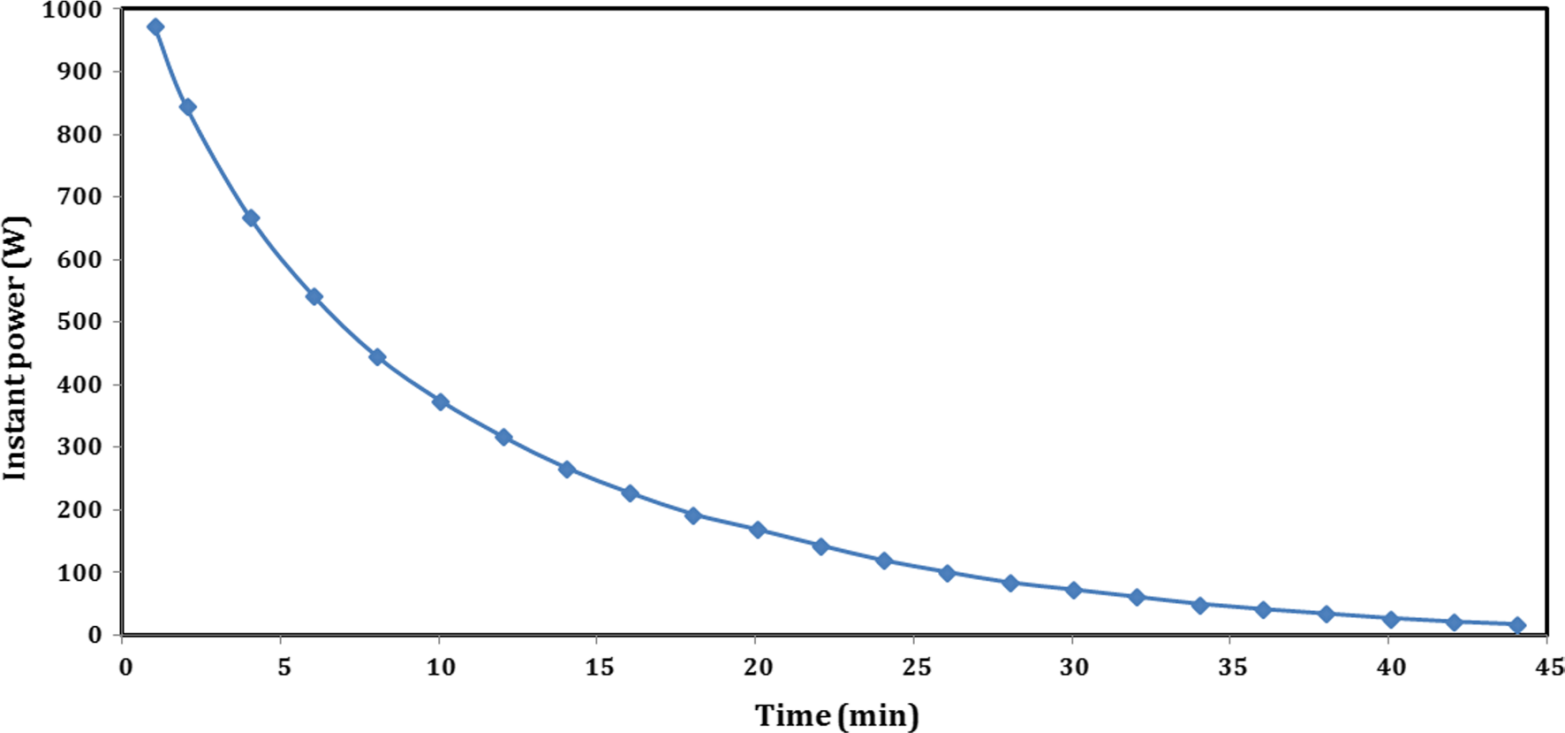
Instant sensible power stored by reactor and sorbent during sensible discharging

As shown in Fig. 10, the trend of stored power was similar to the trend of exit water temperature, which illustrated previously depending on Fig. 9, since the inlet water temperature was constant during the discharging. The instant stored power recorded its highest value of 974.9 W at the beginning and decreased gradually. It can be investigated that the curve can be split into two regions: the first region with sharp decreasing, due to high drop in exit water temperature as large amount of stored heat releasing, as shown in period from 0 to 16 min, where the power decreased to 228.8 W. The second region was from 16 min to the final of the experiment after 44 min, where the power was slightly decreasing as most of stored energy is captured. During this region, the power is decreased until reach its lowest value of 18.5 W. The mean power value was about 253.1 W, and the total power consumed during the sensible heat recovery was 587.2 kJ.

### Temperature and pressure profiles of thermochemical sorption battery

**Fig. 11:**
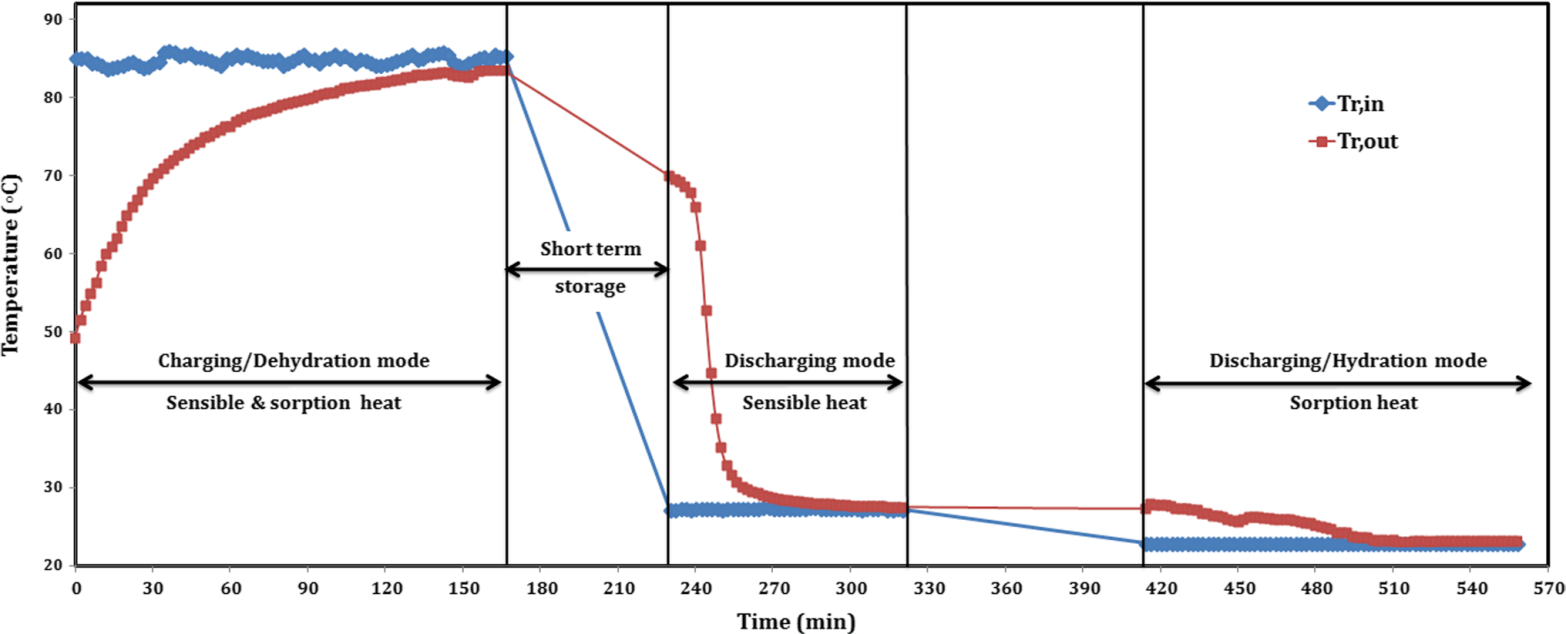
Temperature profile of sorption battery during short-term storage

Regarding short-term storage, combining dehydration and hydration in one figure is shown in Fig. 11. For the total period of 9.3 h, the charging period was about 166 min, sensible heat recovery period was about 90 min, and sorption hydration heat recovery period was about 144 min, with interspersed short-term storage between charging and sensible heat recovery of 64 min. The time lag between ending of the sensible heat recovery and sorption heat recovery was about 94 min. The inlet hot water temperature range was 83.8–86.1 °C, with average dehydration temperature of 85 °C, as mentioned above. The reactor exit water temperature was 49.3 °C, at starting, with temperature difference of 35.9 °C and increased gradually with time. The valve between the reactor and the condenser was closed for the first 44 min, as the composite sorbent did not yet reach the dehydration start temperature of about 69 °C. The difference between inlet and exit reactor water temperatures is decreased gradually and was partly constant through the last 14 min, with ∆T of 2 °C. From 166 to 220 min, the inlet valve of the reactor is closed, since the body of the reactor reached its maximum temperature of about 83 °C, while there was difference between inlet and exit condenser temperatures, which mean that water vapor did not yet totally condense.

During sensible heat recovery, the reactor temperature was controlled by ambient water temperature of 27.4 °C from 230 to 320 min. For real application, the body of the reactor loses sensible heat during the short storage period, assuming a loss of 10 °C temperature difference. Therefore, cold water flows through the reactor to decrease its temperature to about 73 °C, and then the heat recovery is started. After 230 min, the exit reactor temperature was 69.7 °C and decreased sharply to reach 31.7 °C after 254 min, followed by slightly decreasing to its minimum value of 27.5 °C after 320 min. It is worth saying that the difference between inlet and exit reactor temperature was lower than 0.5 °C, from 290 to 320 min, as the reactor body needs long period of time to completely release its sensible heat.

During sorption/hydration heat recovery, the reactor temperature was controlled by water temperature of 23 °C from 414 to 558 min. The valve between the reactor and the evaporator was opened again, to allow liquid water inside the evaporator to take its evaporation heat and flow as adsorbate to the reactor and to hydrate the reactive salt releasing its sorption heat. The exit reactor temperature was 27.3 °C at the beginning, and then it is increased suddenly to 27.9 °C with a maximum difference temperature of about 5 °C during 2 min only. This relatively high temperature difference is established due to the high heat released from the reaction of hydration. However, that did not continue for long period of time, and the difference decreased gradually to reach 3 °C after 56 min from starting and after 470 min in total. After 470 min, the exit reactor temperature decreased gradually and recorded its minimum value of 23.1 °C after 512 min, and then it was relatively remained constant at 32 °C from 502 to 526 min. The exit reactor water temperature recorded a constant value of 23.2 °C, during a period of time of 32 min from 526 to 558 min, with a temperature difference of only 0.2 °C. It is expected that hydration reaction is finished at this time, while the reactor body needed long period of time to transfer its little heat to the working fluid of cold water.

**Fig. 12:**
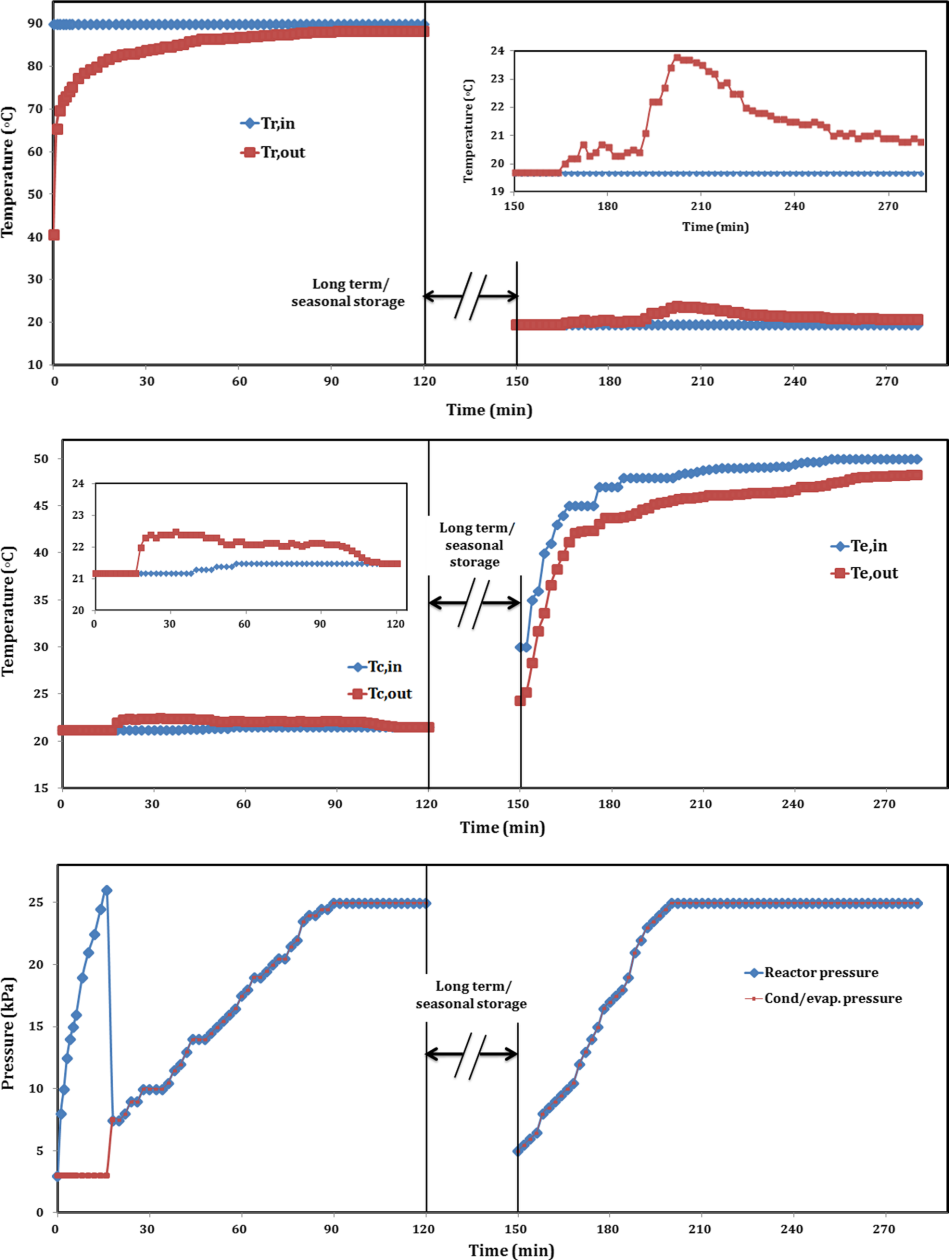
Temperature and pressure profiles of sorption battery during long-term storage

While for long-term/seasonal storage, as shown in Fig. 12. The charging period was 120 min, and sorption hydration heat recovery period was 130 min, with interspersed long-term/seasonal storage between dehydration and hydration of 24 h. The average inlet hot water temperature was 90 °C, as mentioned above. The reactor exit water temperature was 40.7 °C, at starting, with temperature difference of 49.3 °C and increased gradually with time. The valve between the reactor and the condenser was closed for the first 18 min. Therefore, the reactor pressure increased gradually through this time to reach up to 26 kPa and suddenly dropped to 7.5 kPa, while condenser pressure was 3 kPa and suddenly raised to 7.5 when the valve is opened. During this time, inlet and exit condenser temperatures remained constant at 21.2 °C, as shown in Fig. 12. The difference between inlet and exit reactor water temperatures is decreased gradually and was partly constant after 92 min, with ∆T of 1.7 °C. The exit condenser temperature raised once opening the vapor valve and recorded 22.5 °C after 32 min, meaning that high amount of water vapor condensed followed by decreasing in exit temperature up to temperature difference of only 0.1 °C after 108 min. After 120 min, there was no difference between inlet and exit condenser temperatures, meaning that water vapor totally condensed. The pressure of the battery is increased gradually again after opening the valve and reached 24.5 kPa after 86 min. While during 86 and 120 min, the battery pressure kept the same value of 25 kPa. During storage mode, the reactor pressure decreased with time from 25 to 5 kPa, since the reactor released its sensible heat.

During sorption/hydration heat recovery, the reactor temperature was controlled by water temperature of 19.7 °C. At the beginning, the inlet and exit reactor temperature were the same, then the exit temperature increased to 20 °C after 16 min (at 166 min) followed by gradually increments up to 20.6–20.7 °C after 30 min (at 180 min). Evaporation temperature was adjusted at 30 °C, while the pressure was increasing and prevented the liquid water from evaporation. So, evaporation temperature should be increased with the increment of pressure to ensure good evaporation. Sorption process clearly shown after 42 min between 192 and 222 min as shown in Fig. 12 where the exit temperature increased sharply up to its maximum value of 23.8 °C and then decreased gradually up to 22.5 °C after 72 min (at 222 min). As well, the temperature difference decreased gradually after that to reach 1.6 °C after 100 min (at 250 min) and recorded its minimum value of 20.8 °C after 124 min (at 274 min). Exit temperature was relatively remained constant at 20.8–20.9 °C during a period of time of 12 min from 268 to 280 min, with a temperature difference of 1.1–1.2 °C. Through previous experience in the last experiment, we can deduce that the reactor body needed long period of time to transfer its little heat to the cold water as illustrated in Fig. 11 in hydration mode. During hydration, the battery pressure is increased with time due to the water vapor pressure from the minimum value of 5 kPa at the beginning to the maximum value of 25 kPa. The battery pressure remained constant at 25 kPa after 50 min (at 200 min) and remained at this constant value until the end at 280 min as shown in Fig. 12.

### Instant power profile of thermochemical sorption battery

**Fig. 13:**
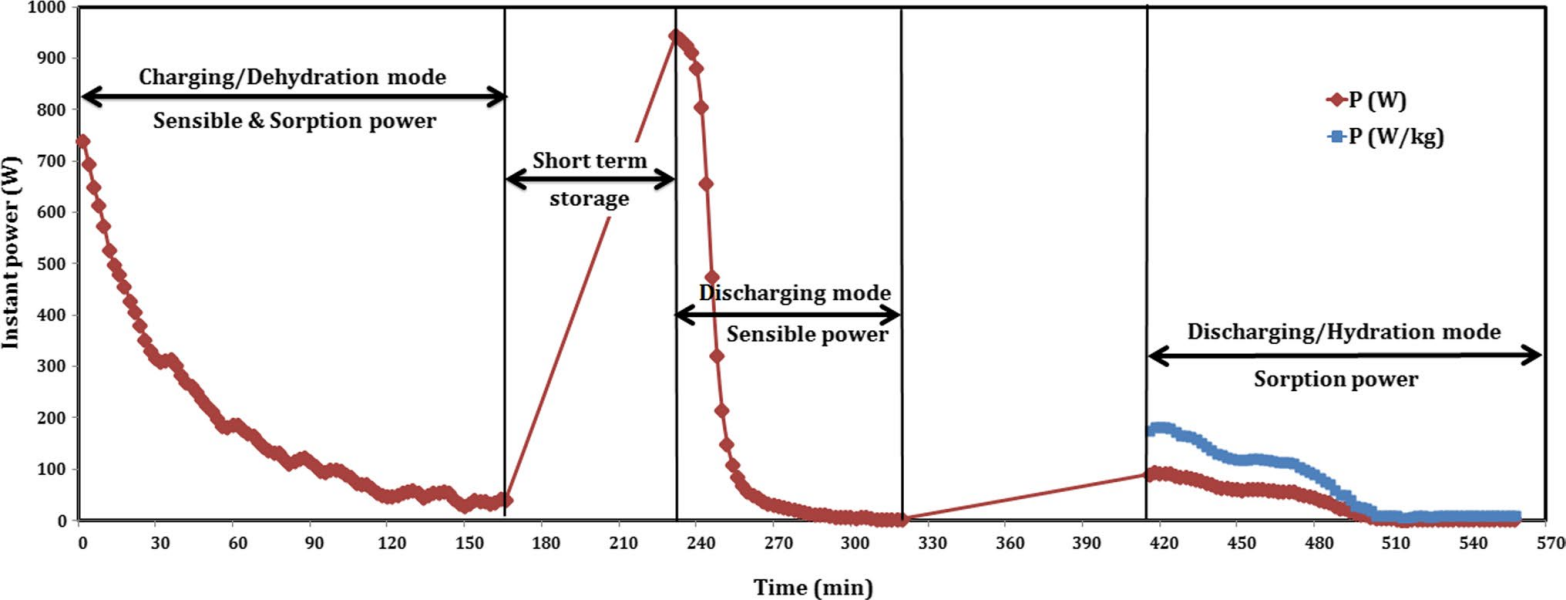
Instant power profile of thermochemical sorption battery during short-term storage

Regarding short-term storage, Fig. 13 presents the profile of the proposed thermochemical sorption battery instant power, with time during charging/dehydration, sensible heat recovery, and hydration/sorption heat recovery of short-term experiment at 85/23 °C since it included sensible heat considerations. The power was derived from the difference between the inlet and exit temperatures. At the beginning of charging (after 2 min), the power recorded its maximum value of 739.3 W and then decreased with time due to increasing the exit reactor temperature. The absorbed power reached 48.9 W after 120 min and then decreased slightly with time to its minimum value of 29.8 W at 150 min. The decreasing of power did not become stable due to the instant effect of unsteady hydration reaction of salt and water vapor. The mean power of charging was about 187.6 W.

At the beginning of sensible discharging period (after 232 min), the power recorded its maximum value of 944.1 W, and then it is decreased with time to its minimum value of 3 W after 320 min, with the same trend as showed in Fig. 13. The mean power of sensible heat recovery was about 178.4 W.

At the beginning of sorption discharging/hydration (after 416 min), the power is recorded to be 89.7 W and then sharply increased to its maximum value of 94.6 W after 418 min, and then it is decreased with time to its minimum value of 2 W after 514 min. During the period from 514 to 526 min, the power ranged between 2 and 3.9 W. While, from 528 min to the end of the proposed experiment after 558 min, the power remained constant at about 3.9 W. The mean power of hydration was 37 W. As mentioned above, the trend of power did not decrease with stable curve due to unsteady hydration heat released that made the exit temperature changed between increase and decrease throw a very little time. As shown in Fig. 13 during sorption power, the value of instant power released per 1 kg of composite sorbent was calculated and plotted with time, and colored with blue above the battery power. The mean power for hydration in case of 1 kg composite sorbent was about 71.2 W for 144 min (169.4 Wh/kg), while this value is very promising in building applications.

**Fig. 14:**
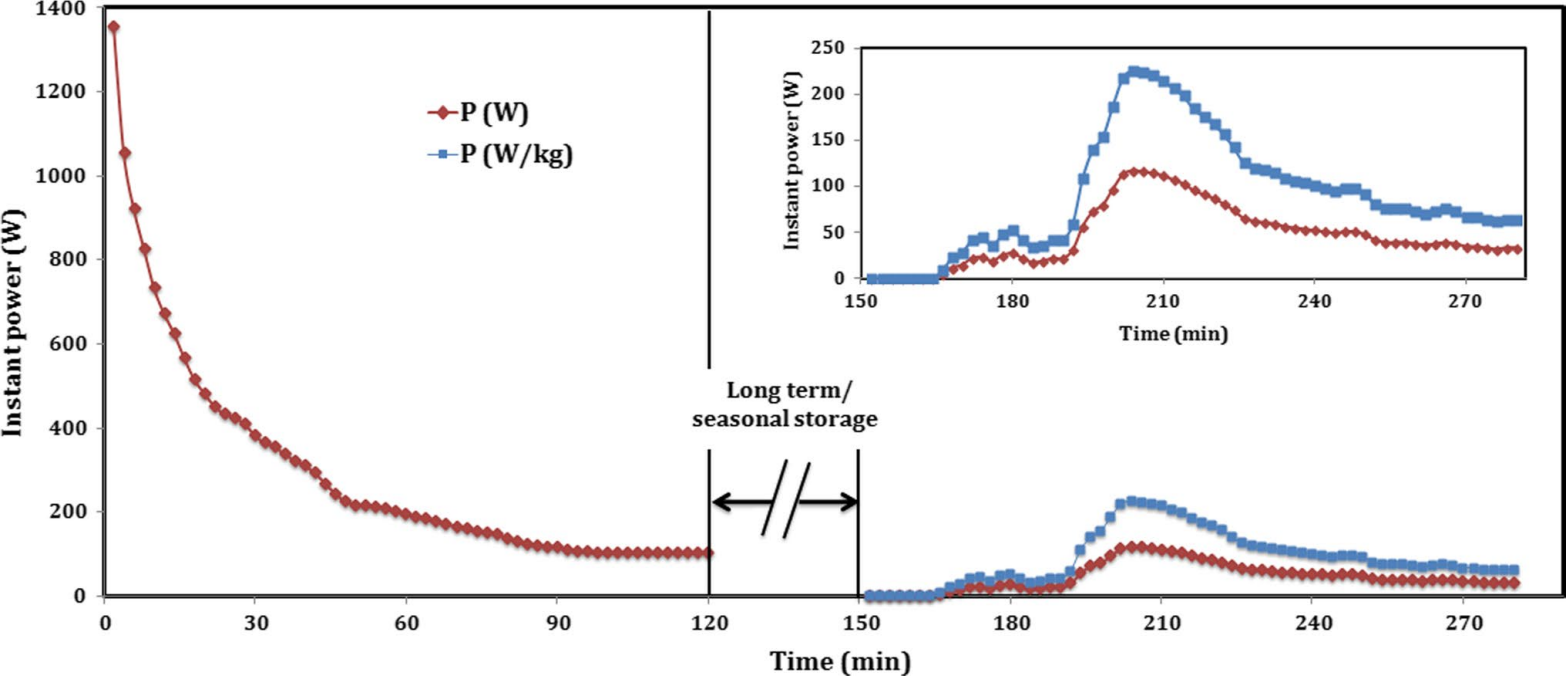
Instant power profile of thermochemical sorption battery during long-term storage

While for long-term/seasonal storage, as shown in Fig. 14. The profile of the instant power, with time during dehydration, and hydration is presented for long-term experiment at 90/19.7 °C. Similarly, at the beginning of charging (after 2 min), the power recorded its maximum value of 1356.1 W and then decreased with time. The input power decreased sharply up to 105.7 W at 94 min and then decreased to its minimum value of 102.7 W during the last 22 min, from 98 to 120 min, while the mean power of charging was about 294.3 W.

After 24 h, at the beginning of sorption heat recovery, there was no power recorded during the first 14 min, and then it is recorded at its maximum value of 4.4 W after 16 min (at 166 min). After that, the output power increased with time to its maximum value of 117.7 W after 54 min (at 204 min) and then decreased gradually up to 36.3 W at 262 min before its unsteady trend between 264 and 280 min with range of 32–39.2 W. The mean power of hydration was 53.7 W. As mentioned above, the trend of power did not decrease with stable curve due to unsteady hydration heat released that made the exit temperature changed between increase and decrease throw a very little time. As shown in Fig. 14 during sorption power, the value of instant power released per 1 kg of composite sorbent was calculated and plotted with time, and colored with blue above the battery power. For long-term storage experiment with dehydration temperature of 90 °C, the mean power for hydration in case of 1 kg composite sorbent was about 103.2 W for more than 130 min (207.9 Wh/kg), while this value is very promising in building applications. Moreover, Table 3 illustrated the important properties and results for the proposed TSES battery.

**Table 3:** Properties and results of TSES battery

Operating mode	T_amb_ (°C)	T_r,in,avg_ (°C)	T_r,in_ (°C)	T_r,start_ (°C)	T_r,final_ (°C)	p_max_ (kPa)	p_min_ (kPa)	*t* (min)	Q (kJ)	Q_out,eff_ (kJ)	ε	P_max_ (W)	P_min_ (W)	P_avg_ (W)	ED_m_ (kJ/kg)	*η* (%)
Sensible charging	38	77	45–80.7	38	75	101	101	56	837.8	-	-	463.3	29.8	247.9	-	-
Sensible discharging	35	35	34.6–36	75	36	101	101	44	587.2	581.7	-	974.9	18.5	253.1	-	69.4
Sorption battery charging at 85 °C	25–26	85	83.8–86.1	25	83	23	5	166	1869.3	-	3	739.3	29.8	187.6	-	-
Sensible heat recovery	27.5	27.4	27.3–27.5	68	27.5	23	5	90	963.1	939.5	3.04	944.1	3	178.4	-	67
Short-term sorption heat recovery	23	23	23	24	23.2	24	5	144	317.1	301.4		94.6	2	37	609.8	64.6
Sorption battery charging at 90 °C	21–22	90	88.2–91.9	21	88	25	3	120	2181.8	-	2.9	1356.1	102.7	294.3	-	-
Long-term sorption heat recovery	19–20	19.7	19–20	19	20.8	25	5	130	389.1	346.3	-	117.7	4.4	53.7	748.3	15.9

### Performance of TSES battery at different charging temperatures

#### Sorption energy density with different charging temperature

**Fig. 15:**
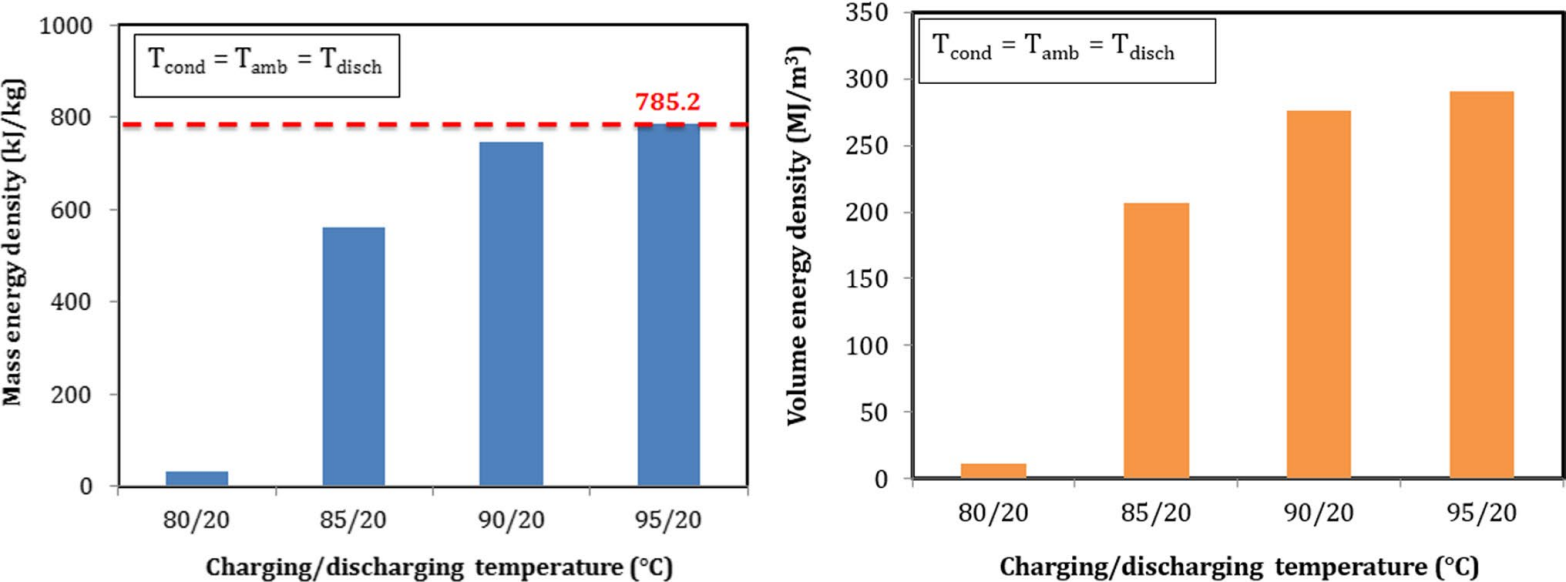
Mass and volume energy densities at different charging temperature

In order to study the capacity of the TSES battery, various charging temperature were used while keeping the condensation and discharging occur at the ambient temperature to present actual sorption results. Mass and volume energy densities can be calculated according to Eqs. (12 and 13). As shown in Fig. 15, mass energy density increased as the charging temperature increased and ranged from a minimum value of 31.7 kJ/kg at 80 °C to a maximum of 785.2 kJ/kg at 95 °C. The same for volume energy density which increased with charging temperature and ranged from a minimum value of 11.7 MJ/m^3^ at 80 °C to a maximum of 290.1 MJ/m^3^ at 95 °C as shown in Fig. 15. For charging temperature of 75 °C, there was no sorption heat released as this temperature could not dehydrate the salt.

Output hydration heat had a large effect on the value of energy density. When the discharging temperatures were at ambient temperature, higher charging temperature aimed to reach a higher global conversion which is the ratio of reacted sorbent to the total sorbent. Synthesis chemical reaction heat increased as the global conversion increased. Charging temperature should be equal the equilibrium desorption temperature or higher to reach a higher global conversion. This was the reason that energy density was very low at 80 °C. We can say that the minimum required charging/dehydration temperature of MgSO_4_ is 85 °C.

#### Energy storage efficiency with different charging temperature

**Fig. 16:**
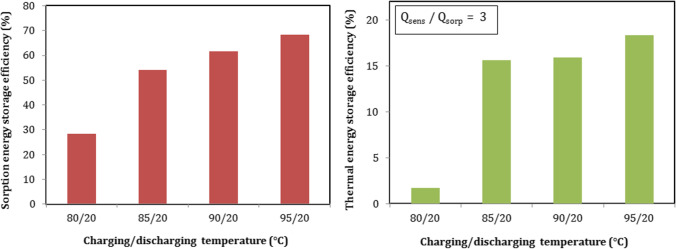
Sorption and thermal energy storage efficiencies at different charging temperature

Thermochemical sorption and thermal energy storage efficiencies can be calculated from Eqs. (14 and 15), respectively. Figure 16 describes the effect of charging temperature on sorption efficiency. As shown in Fig. 16, sorption efficiency increased when the charging temperature increased and ranged from 28.3% at 80 °C to 68.3% at 95 °C. The highest sorption efficiency was 68.3.3% and can be obtained at charging temperature of 95 °C. As mentioned above, the output heat of battery increased with the increase of charging temperature. So, the efficiency recorded the highest value at the highest charging temperature used. The results of total thermal efficiency considering the total input heat (sensible and sorption) are shown in Fig. 16. Taking into account that sensible input heat was 3 times of sorption input heat. Thermal efficiency ranged from 1.7 to 18.3% and recorded its highest value of 18.3% at 95 °C.

### Performance of TSES battery at different discharging temperatures

#### Sorption energy density with different discharging temperature

**Fig. 17:**
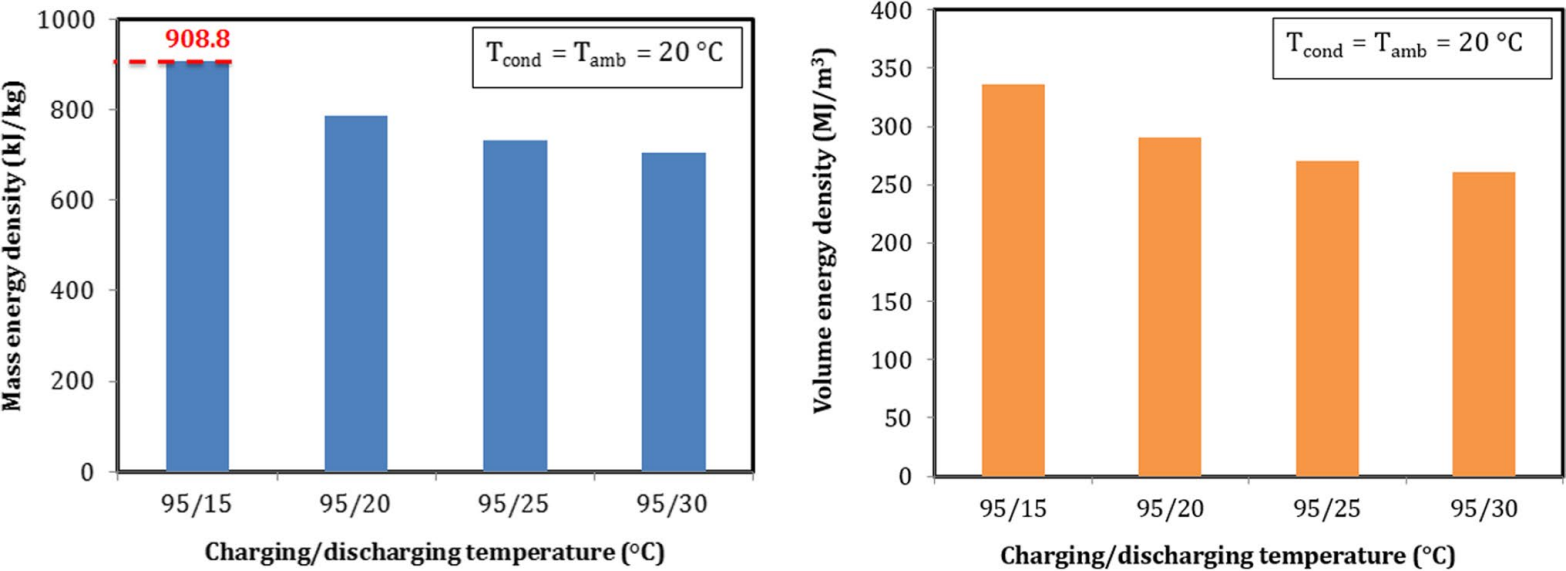
Mass and volume energy densities with different discharging temperature

End users of output heat require various temperatures to meet the different applications. The performance of the thermochemical sorption energy storage is now studied with different discharging temperature. Sorption mass and volume energy densities are shown in Fig. 17. Ambient temperature had a large effect on the results. When discharging temperature was higher than ambient temperature, the heat output affected by losses to the ambient and caused a decrease in energy density and vice versa when the discharge temperature was lower than ambient temperature. As well as, the kinetic of hydration depended on discharging temperature. Lower hydration temperature such as 15 °C accelerated the chemical reaction and caused a higher output heat. Both of energy densities decreased with the increment of discharging temperature. Mass energy density increased from 706.1 to 908.8 kJ/kg, and volume energy density increased from 260.9 to 335.8 MJ/m^3^ when the discharging temperature decreased from 30 to 15 °C. The highest mass energy density was 908.8 kJ/kg and can be achieved at the discharging temperature of 15 °C.

#### Sorption Energy storage efficiency with different discharging temperature

**Fig. 18:**
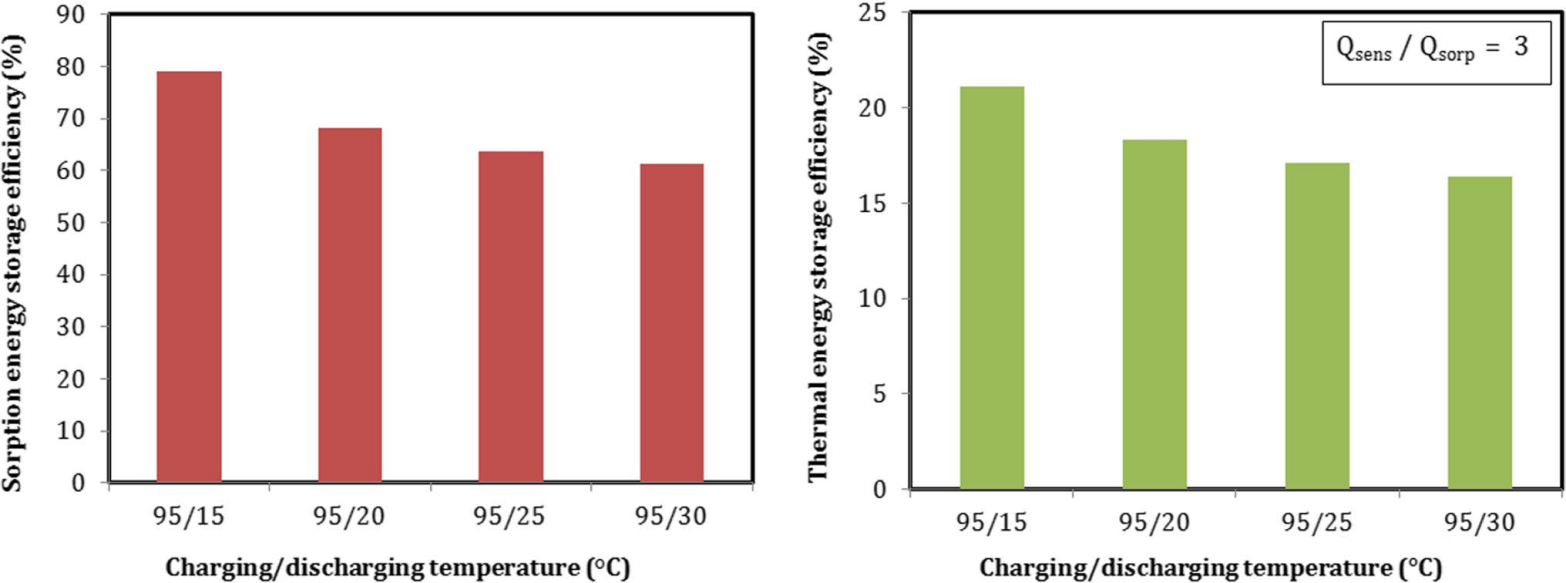
Sorption and thermal energy storage efficiencies with different discharging temperature

Figure 18 describes the effect of discharging temperature on energy storage efficiency when the charging temperature is 95 °C. Sorption efficiency increased when the discharging temperature decreased and ranged from 61.4% at 30 °C to 79.1% at 15 °C. Lower discharge temperature produced higher output heat. Therefore, storage efficiency increased at lower discharge temperature. The highest sorption efficiency was 79.1% at the lowest discharging temperature of 15 °C. The same for the total thermal efficiency, as shown in Fig. 18, and ranged between 16.4 and 21.1%. The maximum thermal efficiency was 21.1% at discharge temperature of 15 °C. Moreover, Table 4 presents a comparison of energy storage systems performance data between this study and the previous studies.

**Table 4:** Comparison of energy storage systems performance data

Working pair	Description	Charging temperature (°C)	Discharging temperature (°C)	Mass energy density (kJ/kg)	References
ENG–TSA/MgSO_4_–H_2_O	Sorption storage	85	23	609.8	This paper
ENG–TSA/MgSO_4_–H_2_O	Sorption storage	90	19.7	748.3	This paper
ENG–TSA/MgSO_4_–H_2_O	Sorption storage	95	20	785.2	This paper
ENG–TSA/MgSO_4_–H_2_O	Sorption storage	95	15	908.8	This paper
ENG–TSA/MgSO_4_–H_2_O	Sorption storage	95	25	733	This paper
ENG–TSA/MgSO_4_–H_2_O	Sorption storage	95	30	706.1	This paper
Zeolite 13X/MgSO_4_/ENG–TSA–H_2_O	Sorption storage	250	25–90	550.8	Xu et al. (2018a)
(ENG–TSA)/SrBr_2_–H_2_O	Sorption storage	90	35	563.1	Zhao et al. (2016)
MgSO_4_/AC–H_2_O	Sorption storage	150	30	920	Nguyen et al. (2023)
MgSO_4_/ HAP–H_2_O	TG-DSC analyses	150	30	464	Nguyen et al. (2022)
EG/MgSO_4_–H_2_O	DSC measurement	150	-	718.9	Miao et al. (2021)
MgSO_4_–Zeolite 13x–H_2_O	DSC measurement	150	-	632	Wang et al. (2019)
Zeolite/mesoporous materials–H_2_O	DSC measurement	117	-	864	Jänchen et al. (2004)
Silica gel/MgSO_4_–H_2_O	STA measurement	150	-	792.7	Ait Ousaleh et al. (2020)
EG/SrBr2/nanocellulose–H2O	DSC measurement	90	23	764	Salviati et al. (2020)
EG/K_2_CO_3_–H_2_O	Sorption measurement	-	25	608.5	Zhao et al. (2022)
MgSO_4_/diatomite–H_2_O	DSC measurement	80–150	25	772.9	Zhang et al. (2021)
EG/SrCl_2_–NH_3_	Sorption storage	94	35	700	Li et al. (2017)
EG/SrCl_2_–NH_3_	Sorption storage	94	55	305	Li et al. (2017)
(ENG–TSA)/MnCl_2_–NH_3_	Sorption storage	175	40	420	An et al. (2022)
EG/MnCl_2_–SrCl_2_–NH_3_	Resorption storage	150	50	444.1	Wu et al. (2019)
(ENG–TSA)/MnCl_2_– CaCl_2_–NH_3_	Resorption storage	150	30	1047	Jiang et al. (2017)
Hydrated salt-based PCC	DSC measurement	50	45-47	184.5	Du et al. (2022)
PA/PVB/EG	DSC measurement	59.5	56.4	128.08–132.87	Lin et al. (2018)
Beeswax-tetradecanol/expanded perlite	DSC measurement	34	29.7	178.7–175.9	Cheng et al. (2018)

*STA* spontaneous thermal analyzer, *DSC* differential scanning calorimeter, *PCC* phase change composite, *PA/PVB/EG* palmitic acid/polyvinyl butyral/expanded graphite

### Performance summary and potential applications of the sorption battery

In previous literature, ENG and ENG–TSA are the most common matrixes which have been used widely, and it can achieve promising results. Here, the porous matrixes of the storage material based on RTEG are proposed with a simple, effective method, more energy-conserving, and pollutant-free approach. RTEG impregnated with MgSO_4_ is suggested as a TSES material for seasonal energy storage although there is no study used such a method to prepare a storage material and used the traditional method of heating NG at high temperature of about 800 °C. Furthermore, the proposed storage material revealed interesting results.

In physical adsorption systems using physical adsorbents such as zeolite, silica gel, activated carbon, and so on, it is normal taking account the density and consequently the amount of sorbent depend on the particle size of the used sorbent. While in chemical adsorption system using active salts such as MgSO_4_, there is a critical issue of agglomeration and swelling of the reactive salt. So, density and amount of salt in composite must be determined carefully to ensure good heat and mass transfer. According to the findings especially for thermal response, the consolidated composite sorbent reached its equilibrium after only less than 1 h. It seems that the proposed RTEG/MgSO_4_ sorbent reveals interesting conductive heat transfer performances.

For mass transfer, although the higher density increases the heat transfer coefficient, but it is more useful for higher mass transfer to kept the adsorbent at lower density especially in the case of low pressure of hydration. The output hydration heat at different conditions compared with the theoretical value that should be achieved according to the number of moles of water adsorbed proves that water adsorption capacity is high. At charging/discharging of 90/20 °C, for example, the theoretical calculated and experimental values are 0.46 and 0.3 g/g, respectively. This proves that the proposed new TSES material with bulk density of 370 kg/m^3^ reveals also interesting mass transfer performances.

Comparing with previous literature, there is no research has been done on using MgSO_4_ as adsorbent, RTEG as porous matrix, and H_2_O as adsorbate for energy storage applications. MgSO_4_ has advantages of low price, availability, high energy density, and moderate equilibrium temperature which can be operated with solar energy of 85–95 °C. Our battery which utilizing hydrate offers benefits over the TSES battery based on ammoniates in terms of safety, affordability, and accessibility. For the costs, as it appears in the configuration of the device, it is low compared to other systems, and the battery can be built with available components. While the battery was working, there was no significant danger.

Comparing with previous studies of sorption/resorption storage especially using salt/NH_3_ which used reactors of shell and tube heat exchanger type, it is a big challenge to use jacket type heat exchanger which has advantages of small area, simple design, easy maintenance, little metals, and little pumped power. In the proposed battery, there is no need for water pump since the circulation occurs with the power of the head. However, the sorption battery could achieve very interesting results.

From the comparison of energy storage systems performance data as in Table 4, most of hydration studies use DSC measurement of little amount of sorbent (~10 mg) to determine the hydration/dehydration heat and the energy storage density. It is a big challenge to produce higher energy capacity using an experimental prototype and to overcome the problems of agglomeration and swelling of salt.

Sensible and latent thermal energy storage suffer from low energy density, unsteady working temperature, low thermal conductivity, susceptible to heat losses, temperature drop of stored thermal energy, phase separation and super-cooling, or insufficient long-term stability of storage capacity. Comparing with them, TSES system has major advantages, including a high energy storage capacity and the possibility of long-term energy retention with negligible heat loss. Therefore, it is why TSES should be employed in the first place compared with other energy storage systems.

For a certain ambient temperature, the energy density, capacity, and output power increase with the increment of charging temperature or the decrease of discharging temperature. The highest output power is 94 kWh/m^3^ and can be obtained for long-term/seasonal storage at charging/discharging temperature of 95/15 °C. Table 5 presented the output of the proposed TSES battery for different conditions.

**Table 5:** Output of the sorption battery for different conditions

Storage	Charging/discharging temperature (°C)	Energy density/capacity/power		
		(kJ/kg)	(Wh/kg)	(kWh/m^3^)
Short-term	85/23	609.8	169.4	63
Long-term	90/19.7	748.3	207.9	77
Long-term	95/20	785.2	218.1	81
Long-term	95/15	908.8	252.4	94

The TSES battery can be used as short-term and/or long-term energy storage for different applications such as solar energy storage, space heating, hot water supply, and industrial heat recovery. Two different sorption experiments were already presented: short-term storage experiment with dehydration temperature of 85 °C and long-term/seasonal storage experiment with dehydration temperature of 90 °C. The potential applications of the sorption battery can be summarized as follows:

- **Short–term energy storage for space heating system**

During charging mode, the reactor is heated with moderate temperature in the daytime, which can be obtained from solar energy and the heat is stored. Discharging mode is divided into sensible and sorption discharging. Sensible heat storage is used for continuous processes to meet with an increase of demand for heat. In the two modes, the reactor supplied with ambient temperature or higher according to the required temperature. During sensible discharging, the valve is closed and the stored sensible heat is released in the reactor. During sorption discharging, the valve is opened and hydration reaction occurs and the heat releases. The output energy can be used for space heating systems to meet the building people’s needs for thermal comfort during night in winter season.

- **Long–term energy storage for domestic hot water system**

During charging mode, the reactor is heated with moderate temperature or higher up to 150 °C, which can be obtained from waste heat source. The heat is stored in form of bonding energy with no heat losses. During discharging mode, sorption discharging mode only is considered and the reactor supplied with ambient temperature. When the heat stored is needed even after months, hydration reaction occurs and the heat releases. The output energy can be used to heat a water tank for a domestic hot water system which distributes hot water to the faucets, shower, tub, and other appliances that people use in the building during winter season.

## Conclusions

Solid–gas thermochemical sorption energy storage (TSES) has a huge attention, due to its advantages of long-term/seasonal storage with much lower or negligible heat loss, high energy storage density/capacity, and high range of steady working temperatures. A promising sorption battery was investigated and analyzed with working pair of composite magnesium sulfate and water vapor. The obtained results showed that the proposed sorption battery is effective for seasonal/long-term energy storage.

The present work presented an interesting material for thermochemical heat storage applications. The proposed new consolidated composite adsorbent, based on expanded graphite treated with sulfuric acid and ammonium persulfate, has major advantages in sorption process and can enhance the performance of dehydration/hydration reaction. The thermal response of the new composite material is studied, and the main conclusions were yielded as follows:

1. During 52 min of charging, the final mean temperature value for the proposed composite material was 75 °C, in case of heating with mean temperature of 77 °C.
2. During sensible discharging, the proposed composite sorbent released its stored energy during only 44 min.
3. It is outstanding for the consolidated composite sorbent to reach its equilibrium after only 52 and 44 min during charging and discharging, respectively.

The thermal effects of reversible reactions between magnesium sulfate and water vapor and the working performance of the proposed TSES battery utilizing composite working pair of RTEG/MgSO_4_–H_2_O are evaluated and analyzed using short-term and long-term storage, and the main conclusions were yielded as follows:

During short-term storage at charging/discharging temperature of 85/23 °C

1. The total heat consumed was 1869.3 kJ and can be divided into sensible and sorption heat. While the total sensible heat during charging was 1402.4 kJ. Hence, the sorption heat consumed for dehydration of 460 g salt and 520 g composite sorbent reached about 466.9 kJ.
2. During sensible discharging of battery, the total output heat released was 963.1 kJ, from the temperature difference of ∆T was 41.5 °C, with thermal efficiency of sensible heat recovery of 67%. While the mean power of sensible heat recovery reached about 178.4 W.
3. During sorption discharging/hydration, the total output heat released was 317.1 kJ, corresponding to energy density of 609.8 kJ/kg composite and 689.4 kJ/kg salt. The thermal efficiency of the sorption heat recovery was 64.6%. While the mean power of hydration in case of 1 kg composite sorbent reached about 71.2 W for 144 min.
4. The total thermal efficiency of battery including sensible and sorption could reach 66.4%.

During long-term storage at charging/discharging temperature of 90/19.7 °C

1. The total heat consumed was 2181.8 kJ and can be divided into sensible and sorption heat. While the total sensible heat during charging was 1620.3 kJ. Hence, the sorption heat consumed for dehydration of 460 g salt and 520 g composite sorbent reached about 561.5 kJ.
2. During sorption discharging/hydration, the total output heat released was 389.1 kJ, corresponding to energy density of 748.3 kJ/kg composite and 845.9 kJ/kg salt. The thermal efficiency of the sorption heat recovery was 15.9%. While the mean power of hydration in case of 1 kg composite sorbent reached about 103.2 W for more than 130 min, and this value is very promising in building applications.

Performance of TSES battery at different charging and discharging temperatures is studied. The main conclusions were yielded as follows:

1. The minimum required dehydration temperature of MgSO_4_–H2O was 85 °C.
2. The experimental results show that energy storage density and sorption efficiency increase with the increment of charging temperature or decreasing of discharging temperature at a certain ambient temperature.
3. Under experimental conditions, energy density ranged from 31.7 to 908.8 kJ/kg (corresponding to volume energy density from 11.7 to 335.8 MJ/m^3^) where the highest energy density was 908.8 kJ/kg when charging, condensation, and discharging temperatures were 95, 20, and 15 °C, respectively.
4. Sorption energy efficiency ranged from 28.3 to 79.1% under experimental conditions where the highest value obtained at charging/discharging temperature of 95/15 °C. The maximum thermal efficiency was 21.1% at charging/discharging temperature of 95/15 °C with sensible to sorption ratio of 3:1.

By improving the system design, the performance of the sorption battery may be increased. So, future research should put several works into developing thermochemical sorption energy storage for use in practical applications. Future directions can be summarized as:

1. The study is focused on heat storage capacity. However, a major interest of such storage material is the heat and mass transfer properties.
2. Studying the performance of the proposed storage material under different bulk density, salt solution ratio, salt to matrix ratio, and so on can be done to choose the best properties of the material.
3. The sorption results of battery can be enhanced by increasing the diameter of the connecting pipe between the reactor and the condenser/evaporator, decreasing the weight of the battery, increasing the amount of sorbent.
4. Increasing the charging temperature increases the storage capacity. Dehydration temperature up to 150 °C can be studied.

## Data Availability

The datasets used and/or analyzed during the current study are available from the corresponding author on reasonable request.

## Nomenclature

AC: Activated carbon
ATB: Adsorption thermal battery
C_p_: Specific heat at constant pressure (kJ/kg∙K)
DRH: Deliquescence relative humidity
ED: Energy density (kJ/kg)
EG: Expanded graphite
ENG: Expanded natural graphite
ENG–TSA: Expanded natural graphite treated with sulfuric acid
HAP: Hydroxyapatite
LHS: Latent heat storage
ṁ: Mass flow rate of heat transfer fluid (kg/s)
m: Mass (kg)
P: Power (W)
p: Pressure (kPa)
PCM: Phase change material
Q or q: Heat amount/quantity (kJ)
RH: Relative humidity
RTEG: Room temperature expanded graphite
SHS: Sensible heat storage
T: Temperature (k or °C)
t: Time (s or min)
TES: Thermochemical energy storage
TSES: Thermochemical sorption energy storage
V: Volume (m^3^)

## Greek symbols

∆H: Reaction enthalpy (kJ/mol)
∆T: Temperature difference (K)
∆t: Time interval (s)
*ε*: Ratio of sensible to sorption heat
*η*: Efficiency (%)

## Subscripts

avg: Average
c: Condensation
cs: Composite sorbent
e: Evaporation
out,eff: Effective heat output
in: Inlet/input
m: Mass
max: Maximum
min: Minimum
out: Outlet/output
r, in: Inlet of chemical reactor
r, out: Outlet of chemical reactor
sens: Sensible
sorp: Sorption
th: Thermal
